# Coriander (*Coriandrum sativum* L.) essential oil and oil-loaded nano-formulations as an anti-aging potentiality via TGFβ/SMAD pathway

**DOI:** 10.1038/s41598-022-10494-4

**Published:** 2022-04-21

**Authors:** Mohamed A. Salem, Eman G. Manaa, Nada Osama, Nora M. Aborehab, Mai F. Ragab, Yusuf A. Haggag, Magda T. Ibrahim, Dalia I. Hamdan

**Affiliations:** 1 Department of Pharmacognosy and Natural Products, Faculty of Pharmacy, Menoufia University, Gamal Abd El Nasr St., Shibin Elkom, Menoufia 32511 Egypt; 2 Clinical Pharmacy Department, Shibin Elkom Teaching Hospitals, Gamal Abd El Nasr St., Shibin Elkom, Menoufia 32511 Egypt; 3 Biochemistry Department, Faculty of Pharmacy, Menoufia University, Gamal Abd El Nasr St., Shibin Elkom, Menoufia 32511 Egypt; 4 Department of Biochemistry, Faculty of Pharmacy, October University for Modern Sciences and Arts (MSA), Giza, 12451 Egypt; 5 Pharmacology Department, School of Life and Medical Sciences, The University of Hertfordshire Hosted By Global Academic Foundation, New Administrative Capital, Cairo, Egypt; 6 Department of Pharmaceutical Technology, Faculty of Pharmacy, Tanta University, Tanta, Egypt; 7 Department of Pharmacognosy, Faculty of Pharmacy, Al-Azhar University, Cairo, Egypt

**Keywords:** Pharmaceutics, Pharmacology

## Abstract

Aging has become a concern for many people, especially women. Given that high-quality anti-aging products are of high cost; it has imperative to search for other economical sources. Essential oils are frequently used in cosmetics products due to a wide range of biological activities as well as their pleasant odor. The current study aimed to investigate the biochemical effect of the cosmetic potential of selected Apiaceous essential oils, traditionally used for skincare, by evaluating their anti-wrinkle activity. It is worth noting that, coriander essential oil showed the highest collagenase, elastase, tyrosinase, and hyaluronidase inhibitory activities compared to other Apiaceous oils (fennel, anise, and cumin). GC–MS proved that coriander essential oil showed a very high level of oxygenated monoterpenes, with linalool (81.29%) as the most abundant constituent. Intriguingly, coriander oil cream and Coriander Essential Oil-loaded Lipid Nanoparticles (CEOLNs) formulations attenuated in vivo UV-induced skin photoaging that was manifested by significantly decreased MDA, COX-2, PGE-2, MMP-1, JNK, and AP-1 levels. Moreover, these pharmaceutical dosage forms significantly increased skin collagen content compared to UV-injured group. Also, coriander essential oil significantly increased TGFβ, TGFβII, and SMAD3 protein expression levels compared to UV-injured group. In conclusion, the pharmaceutical dosage forms of coriander oil possess anti-wrinkle activities that could have an auspicious role in amending extrinsic aging.

## Introduction

The skin is provided with sensory and computing skills to combat environmental stimuli to sustain and repair disrupted cutaneous homeostasis. A cutaneous neuro-endocrine system coordinates these complicated processes, as well as communicating bidirectionally with the central neurological, endocrine, and immunological systems, all of which work together to maintain bodily homeostasis^1^. The epidermis, the skin's most superficial layer, is generated from the ectoderm and is marked by continual renewal. Keratinocytes are the epidermis' major constituents, together with other cells whose function is more regulatory than structural; melanocytes are derived from the neural crest. Melanin is a protective pigment generated by these cells which are transmitted from melanocytes to keratinocytes via apocopation^2^. Epidermal melanin has a profound evolutionary and physiological significance. As a result of its optical and chemical filtering characteristics, high melanin content (racial pigmentation) protects the skin from UV-induced skin damage^3^.

Aging is the progressive loss of an organism’s homeostatic balance^4^. One of the most common dermatological issues is skin aging. Degradation of the extracellular matrix (ECM) occurs in the epidermal and dermal layers of the skin, resulting in visible signs on the skin's surface and changes in the skin's physical characteristics^5^. Skin aging is a complicated biological process impacted by a mix of internal (chronological aging) and external (photoaging) aspects^6^. Intrinsic aging occurs due to genetics, cellular metabolism, and hormonal imbalance factors. Where the extrinsic aging may be attributed to chronic exposure to Ultraviolet radiation (UVR), contamination, ionizing, radiation, chemicals, and toxicants issues^6^. Photoaging is characterized by dry, rough, colored, and oxidized skin, particularly on the face and hands. On the other hand, fine and smooth wrinkles on dry pale skin give the appearance of intrinsic aging^7^.

Exposure to UVR is the main reason for the oxidative stress in the skin, making it a significant risk factor for the development of skin disorders such as wrinkles, lesions, and cancer^7^. Reactive oxygen species (ROS) induces skin aging by causing oxidative damage to the skin's lipids, proteins, and DNA. Matrix metalloproteinases (MMPs) production can also be induced indirectly by ROS via the mitogen-activated protein kinase (MAPK) pathway^8^. MMP-1, also known as interstitial collagenase, is a protein that starts the breakdown of collagen types I, II, and III in the skin. The degradation of collagen and other ECM proteins is caused by the upregulation of UV-induced MMPs in dermal fibroblasts^9^. Collagenase is responsible for ECM reconfiguration which includes collagen degradation. Elastase, a serine proteinase, is the enzyme that breaks down elastin in the ECM. As collagen and elastin are primarily responsible for maintaining skin structural integrity and elasticity, their loss contributes to undesirable wrinkles and aging skin^8^.

Additionally, skin aging may be influenced by nutritional supplements. Antioxidant-rich foods, such as vitamin C and a high vegetable intake, as well as olive oil, have been linked to a less wrinkled appearance, but higher fats and carbohydrates intake have been linked to a higher risk of wrinkled appearance^10^. Moreover, wrinkles are thought to be caused by too much sugar in the bloodstream, which interacts with proteins and forms hazardous new molecules known as advanced glycation end products (AGEs)^10^. Besides this, the more sugar intake, the more AGEs acquired, and the more glycation occurs. AGEs deposits in fibronectin, laminin, elastin, and collagen are found in the skin^10^. After 35 years of age, glycation appears in the dermis, and UV exposure enhances cross-linking in the skin^10^.

Sun avoidance and the use of sunscreens (to block or reduce skin exposure to UV radiation), retinoids (to inhibit collagenase synthesis and enhance collagen production), and anti-oxidants as oral supplements or topical therapies are the known strategies for preventing photo-aging^11^. Furthermore, plastic surgery and laser rejuvenation treatments, which are considered modern science and technology, are associated with risks and complications. In recent years, the rising demand for pharmaceutical and cosmetic preparations containing natural active ingredients has directed researchers' attention to essential oils (EOs)^12^. Plants from the Apiaceae family are a potential source of medicines such as essential oils, terpenoids, triterpenoid saponins, flavonoids, coumarins, polyacetylenes, and steroids^13^. The essential oils obtained from the fruits of this family have anti-tumor, antiulcer, antimicrobial, antioxidant, anti-inflammatory, antispasmodic, antiseptic, and antiwrinkle effects^13^.

The most frequent Apiaceous members used traditionally include Fennel, Anise, Cumin, and Coriander^14^. Traditionally, consumers believe that the essential oils from the previous plants give the skin the deep cleaning it needs, and therefore it works to clean the pores and prevent clogging, which works to whiten the skin. The essential oils are also cleaning the skin surfaces from hyperpigmented spots and impurities that appear on them. Accordingly, in this work, the essential oils of the four selected plants (fennel, anise, cumin, and coriander) were tested in vitro for their anti-aging potential. GC–MS profiling of the selected Apiaceous oils has corroborated the results of in vitro study which showed that coriander oil had the highest antiwrinkle activities. Therefore, an in-vivo evaluation of an anti-aging effect of coriander oil dosage forms against UV radiation-induced wrinkles was evaluated and exhibited promising activities. The current study was the first to report anti-skin-aging activity of coriander oils Nano-formulations.

## Results

### In vitro screening of selected oils on skin aging-related enzyme activities

#### Elastase enzyme

**Figure 1:**
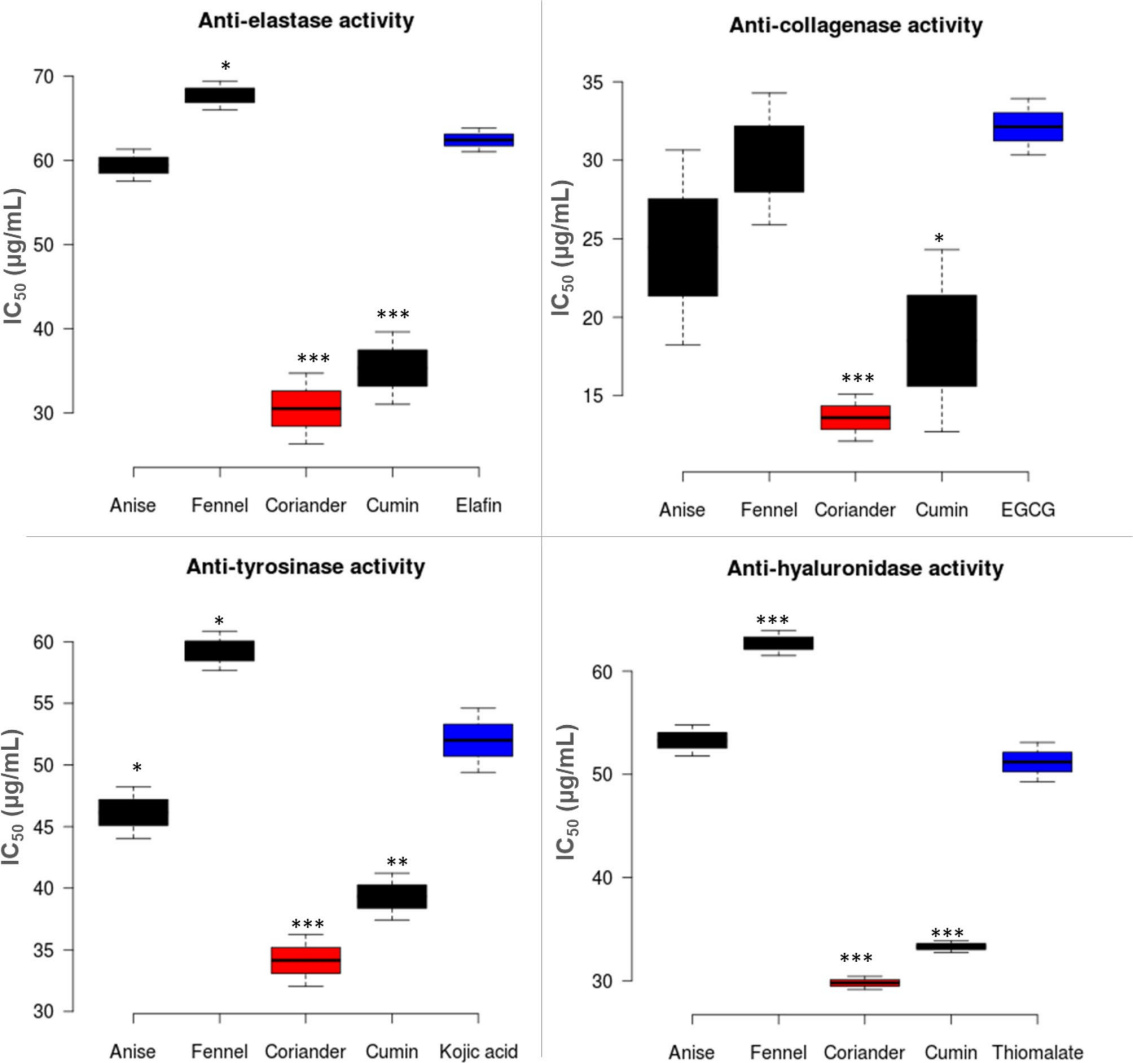
Anti-elastase, anti-collagenase, anti-tyrosinase and anti-hyaluronidase activities of selected Apiacaeae essential oils. The results are expressed as the mean ± SD, n = 3. Asterisks indicate significant differences from the standard drug (*, **, ***, *p* < 0.05, *p* < 0.01, *p* < 0.001, Student’s t test).

Elastase activity is based on the release of p-nitroaniline from the substrate N-succinyl-Ala-Ala-Ala-p-nitroanilide. The inhibitory effects of Fennel, Anise, Cumin, and Coriander Essentail oils (EO) on Elastase enzyme were evaluated as illustrated in Fig. 1. Coriander oil showed the highest inhibition activity (IC_50_ = 30.5 ± 4.2 µg/mL) followed by the oil of cumin, anise and, fennel (IC_50_ = 35.31 ± 4.3, 59.4 ± 1.9 and 67.7 ± 1.7 µg/mL), respectively, when compared to the standard elafin (IC_50_ = 62.4 ± 1.4 µg/mL) (Fig. 1).

#### Collagenase enzyme

Collagenase activity was determined with a spectrofluorimetric method using a fluorogenic metalloproteinase-2 (MMP2) substrate (MCAPro-Leu-Ala-Nva-DNP-Dap-Ala-Arg-NH2) which is enzymatically degraded by collagenase to produce fluorescence**.** The summarized inhibitory potential of the tested EO against tyrosinase was demonstrated in Fig. 1. Coriander oil showed the strongest inhibition activity of the collagenase enzyme with IC_50_ = 13.6 ± 1.5, followed by the oil of cumin (IC_50_ = 18.5 ± 5.8 µg/mL). Moreover, oil of fennel and anise displayed significant activity with (IC_50_ = 30.08 ± 4.2 and 24.45 ± 6.2 µg/mL), respectively, when compared to the standard d epigallocatechin gallate (EGCG) (IC_50_ = 32.13 ± 1.8 µg/mL) (Fig. 1).

#### Tyrosinase enzyme

The anti-tyrosinase activity of the selected oils was evaluated through the conversion of L-DOPA to dopaquinone by mushroom tyrosinase. The results indicated that coriander oil showed the highest inhibitory activity agaisnt tyrosinase (IC_50_ = 34.14 ± 2.1 µg/mL) compared to the standard kojic acid (IC_50_ = 52.0 ± 2.6 µg/mL) (Fig. 1). Oil of cumin, anise, and fennel (IC_50_ = 39.32 ± 1.9**,** 46.14 ± 2.1and 59.25 ± 1.6 µg/mL) showed lower inhibitory activity than coriander. Fennel EO showed the lowest activity when compared to other oils and the standard drug.

#### Hyaluronidase enzyme

The effects of selected oils on hyaluronidase activity indicated that coriander and cumin oils showed the highest inhibition activity (IC_50_ = 29.79 ± 0.63 and 33.31 ± 0.58 µg/mL, respectively) compared to the standard sodium aurothiomalate with (IC_50_ = 51.2 ± 1.9 µg/mL) (Fig. 1), but still cumin is less active than coriander oil. However, anise (IC_50_ = 53.3 ± 1.5) and fennel EO (IC_50_ = 62.72 ± 1.2 µg/mL) showed the lowest anti-hyaluronidase activity when compared to other oils and the standard drug.

### Chemical composition of the essential oils

GC and GC–MS were used to identify 53 components from the essential oil of four Apiaceous fruits, as shown in Table 1. GC analysis of the four oils showed that oxygenated monoterpenes in fennel oils accounted for 80.19% of the total compounds detected. This is attributed mainly due to Methyl chavicol (79.88%). Coriander oil showed a very relatively high concentration of oxygenated monoterpenes (90.38%), which was mostly attributable to linalool (81.29%). While in Cumin oil, both monoterpenes and oxygenated monoterpenes represented the main constituents representing 39.66 and 59.3%, respectively. Anise oil exhibited a high concentration of oxygenated monoterpenes, representing 92%, owing to anethole (91.8%).

**Table 1:** Volatile oil constituents identified in the essential oils of fennel, anise, coriander, and cumin fruits were analyzed by GC–MS.

Compounds	(RI)	Rel. abundance (%)				IM
		Fennel	Anise	Coriander	Cumin	
*α*-Pinene	939	1.58	–	**4.58**	0.72	RI, MS
Camphene	954	0.04	–	0.48	–	RI, MS
Sabinene	954	0.34	–	0.16	0.8	RI, MS
*β*-Pinene	979	0.14	–	0.39	**14.43**	RI, MS
Myrcene	990	0.2	–	0.13	0.53	RI, MS
Carene	1002	0.07	–	–	0.07	RI, MS
Cymene	1024	0.15	–	0.49	**4.56**	RI, MS
Limonene	1029	**12.5**	–	1.05	–	RI, MS
1,8-Cineole	1031	0.18	–	–	0.31	RI, MS
*O*-Cimene	1037	0.37	–	–	–	RI, MS
Terpinene	1059	0.19	–	**3.64**	**17.33**	RI, MS
Camphor	1146	0.1	–	**3.61**		RI, MS
Methyl chavicol	1196	**79.88**	–	–		RI, MS
Fenchyl acetate	1220	0.1	–	–	–	RI, MS
*α*-Copaene	1376	0.03	–	–	–	RI, MS
Germacrene	1481	0.03	–	–	–	RI, MS
Anethole	1284	–	**91.8**	–	–	RI, MS
Element	1338	–	0.23	–	–	RI, MS
*α*-Himachalene	1451	–	0.43	–	–	RI, MS
*β*-Himachalene	1482	–	**5.33**	–	–	RI, MS
*α*-Longipinene	1352	–	0.04	–	–	RI, MS
Cyclosativene	1371	–	0.02	–	–	RI, MS
*α*-Ylangene	1375	–	0.07	–	–	RI, MS
*β*-Elemene	1390	–	0.09	–	–	RI, MS
*α*- zingiberene	1493	–	0.75	–	–	RI, MS
*β*- Himachalene	1500	–	0.31	–	–	RI, MS
*β*-Bisabolene	1505	–	0.25	–	–	RI, MS
*β*-Sesquiphellandrene	1522	–	0.06	–	–	RI, MS
*α*-Thujene	930	0.02	–	0.03	0.22	RI, MS
Terpinolene	1088	–	–	0.13	–	RI, MS
Linalool	1096	–	–	**81.29**	–	RI, MS
Citronellal	1153	–	–	0.14	–	RI, MS
Borneol	1169	–	–	1.48	–	RI, MS
*α*-Phellandrene	1002	–	–	–	0.34	RI, MS
Sylvestrene	1030	–	–	–	0.66	RI, MS
Terpinene-4-oL	1177	0.03	–	0.22	0.08	RI, MS
*β*-cyclocitral	1219	–	–	–	1.12	RI, MS
Cumin aldehyde	1241	–	–	–	**16.36**	RI, MS
*α*-Terpinene-7-oL	1285	–	–	–	8	RI, MS
*β*-Terpinene-7-oL	1291	–	–	–	**32.96**	RI, MS
Mentha-14-dien-7-oL	1327	–	–	–	0.47	RI, MS
Daucene	1381	–	–	–	0.15	RI, MS
Caryophyllene	1408	–	–	–	0.15	RI, MS
*β*-Farnesene	1442	–	–	–	0.14	RI, MS
*β*-copaene	1432	–	–	–	0.06	RI, MS
*α*-Acoradiene	1466	–	–	–	0.11	RI, MS
Carotol	1594	–	–	–	0.28	RI, MS
Eicosane	2666	–	–	0.57	–	RI, MS
Tetratetra acontane	2851	–	–	0.53	–	RI, MS
Heneicosane	2368	–	–	0.34	–	RI, MS
Hexatriacontane	2666	–	–	0.41	–	RI, MS
Hexadecanoic acid, trimethylsilyl ester	2022	0.02	0.02	–	0.03	RI, MS
**Total area**		**95.97%**	**99.40%**	**99.67%**	**99.88%**	
**Monoterpene hydrocarbons**		**15.6%**	**–**	**7.44%**	**39.66%**	
**Oxygenated monoterpenes**		**80.19%**	**91.8%**	**90.38%**	**59.3%**	
**Sesquiterpene hydrocarbons**		0.06%	**7.58%**	–	0.61%	
**Oxygenated sesquiterpenes**		0.1%	–	–	0.28%	
**Others**		0.02%	0.02%	1.85%	0.03%	

Significant values are in bold.

### Preparation and characterization of CEOLNs

The promising in vitro anti-wrinkles activity, as well as GC–MS analysis of the essential oils prompted us to formulate the coriander oil in a pharmaceutical form. This part aims to design and formulate two different types of lipid nanoparticles encapsulating coriander essential oil. CEOSLNs and CEONLC were prepared to entrap the oil inside the matrix of different lipid nanosystems. The used oil is unstable as it is easily volatilized and can be degraded by oxidation, Therefore, nanoencapsulation of this oil into lipid nanosystems represents a promising tool to enhance its stability and improve its therapeutic activity^12,15^. Herein, we prepared oil-loaded lipid nanoparticles based on natural solid and liquid lipids of cocoa butter and olive oil. Lecithin was optimally chosen as a surfactant to stabilize the formulated nanoparticles and to prevent their aggregation. The lipid phase including only cocoa butter resulted in the fabrication of SLNs. On the other hand, the lipid phase composed of cocoa butter and olive oil resulted in the formulation of NLC encapsulating the oil^15^.

Given these formulation conditions, the physicochemical properties of the prepared CEO-loaded lipid nanoparticles were demonstrated in Table 2. The nanosize range of the prepared lipid nanosystems was 138.33 ± 18.66–317.91 ± 35.89 nm. CEOSLNs showed a significantly bigger size compared to NLC (p ˂ 0.01). CEONLC exhibited a lower PDI value compared to oil extract-loaded SLNs (p˂ 0.05). The lower PDI value indicates the homogenous size distribution of the prepared nanocarriers. In addition, the higher zeta potential value of NLC demonstrates a high degree of in vitro stability against aggregation.

**Table 2:** Physicochemical properties  of the prepared Coriander oil-lipid nanoparticles.

Formula	Physicochemical characterizations		
	Size (nm)	PDI	Zeta potential (− mV)
Blank SLNs	245.24 ± 24.25	0.273 ± 0.054	− 19.47 ± 3.78
Blank NLS	138.33 ± 18.66	0.314 ± 0.047	− 21.47 ± 5.24
CEOSLNs	317.91 ± 35.89	0.335 ± 0.085	− 23.014 ± 4.49
CEONLC	175.24 ± 24.25	0.245 ± 0.011	− 27.47 ± 3.78

Results are represented as mean ± SD for (n = 3).

**Figure 2:**
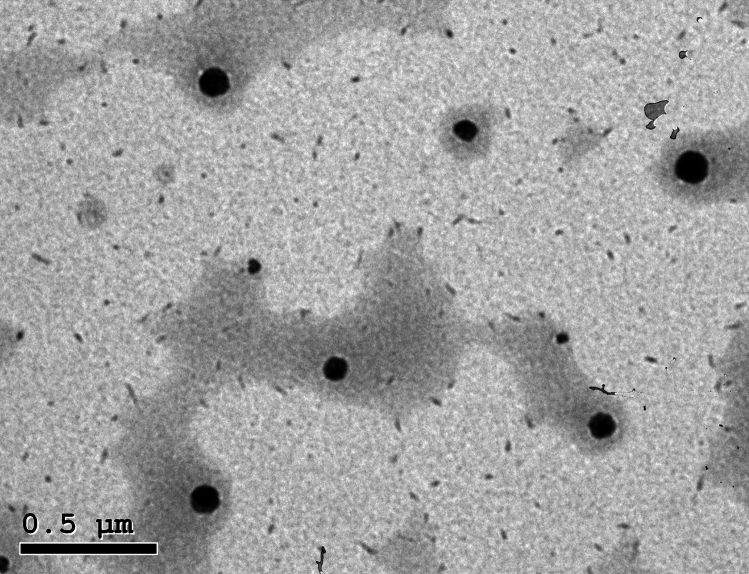
TEM image of CEONLC nanoemulgel.

As shown in Fig. 2, oil-loaded NLC showed a spherical and homogenous size appearance with low nanosize under the microscope. These results are in good agreement with particle size and PDI results obtained from the zetasizer. According to the previous results, CEONLC was chosen as an optimum formulation for nanoencapsulation of oil depending on its significantly lower size, PDI, and higher zeta potential. Oil-loaded NLC was used for the formulation of nanoemulgel as a final dosage form. The prepared nanoemulgel was used for topical application in the *in-vivo* study.

### In vivo anti-wrinkles activity of coriander oil formulas

#### Oxidative stress biomarkers levels

**Figure 3:**
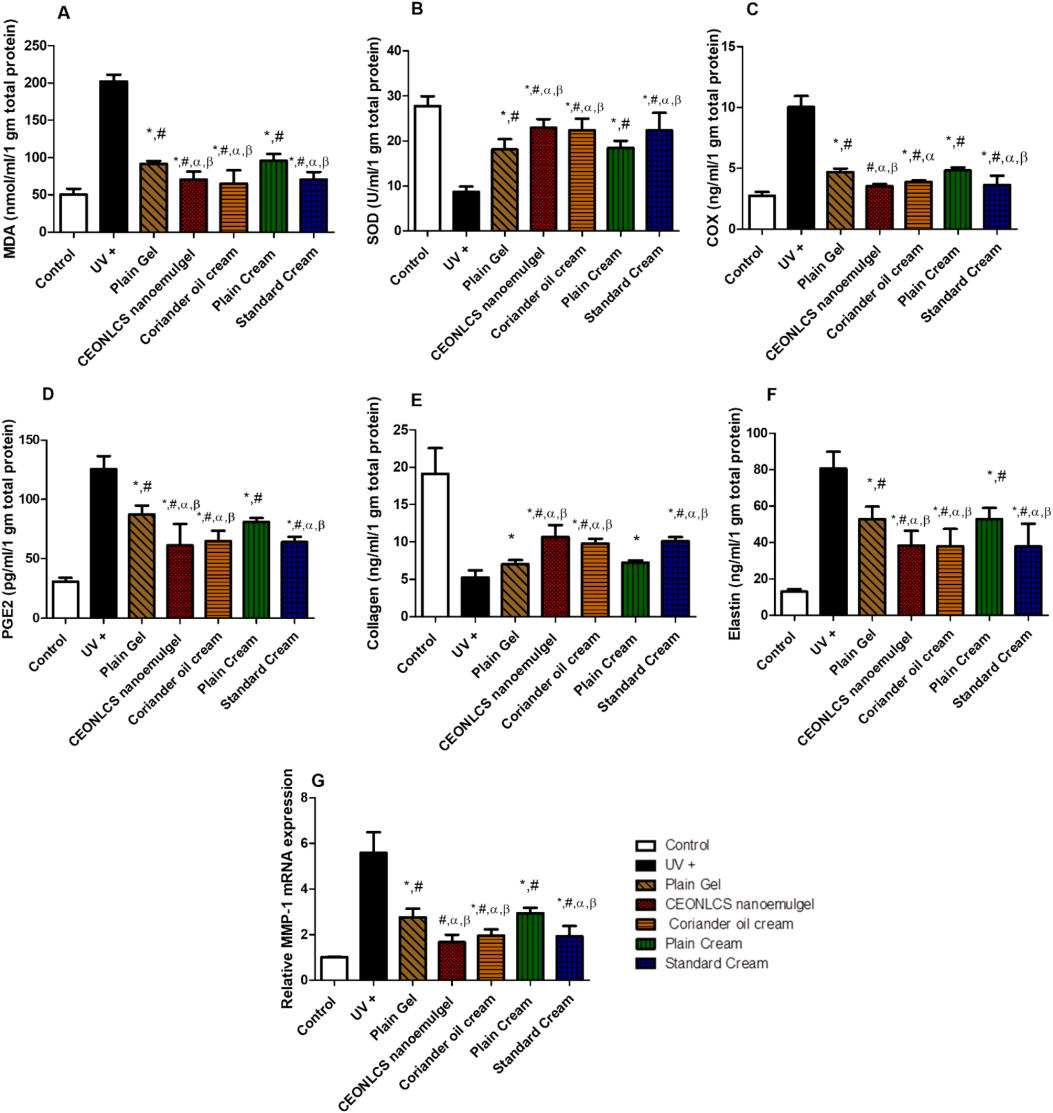
Effect of plain cream, plain gel, Coriander oil cream formula, CEONLC nanoemulgel formulation, and standard cream treatment on levels of (**A**) MDA, (**B**) SOD, (**C**) COX-2, (**D**) PGE-2, (**E**) Collagen, (**F**) Elastin and MMP-1 (**G**), in skin homogenates of photoaged mice. Each result represents the mean value for 8 mice ± SD of the mean. Statistical analysis was carried out by one-way ANOVA followed by Tukey’s multiple comparison test. *Statistically significant from the normal control group at *p* ≤ 0.05, ^#^statistically significant from the UV injured group at *p* ≤ 0.05, *α* statistically significant from the plain cream base group at *p* ≤ 0.05, and *β* statistically significant from the plain gel base group at *p* ≤ 0.05.

UV irradiation led to increased reactive oxygen species production which was reflected in a significantly increased level of Malondialdehyde (MDA) (202.1 ± 9.02 nmol/mL/1 gm total protein) and significantly decreased level of superoxide dismutase (SOD) (8.69 ± 1.19 U/mL/1 gm total protein) in UV- injured group as compared to the control (MDA: 50.28 ± 7.94 nmol/mL/1 gm total protein; SOD: 27.76 ± 2.17 U/mL/1 gm total protein) at *p* < 0.05. Coriander oil cream (MDA: 65.10 ± 17.95 nmol/mL/1 gm total protein; SOD: 22.37 ± 2.58 U/mL/1 gm total protein) and CEONLS nanoemulgel formulation (MDA: 70.47 ± 10.98 nmol/mL/1 gm total protein; SOD: 22.96 ± 1.88 U/mL/1 gm total protein) attenuated ROS production by UV as manifested in significantly decreased level of MDA and increased level of SOD as compared to UV injured group at *p* < 0.05 (Fig. 3).

#### Cyclooxygenase 2 (COX-2) tissue level

UV irradiation significantly increased level of COX-2 (10.04 ± 0.91 ng/mL/1 gm total protein) as compared to control (2.76 ± 0.30 ng/mL/1 gm total protein) at *p* < 0.05. Coriander oil in both cream (3.88 ± 0.13 ng/mL/1 gm total protein) and CEONLC nanoemulgel formulation (3.54 ± 0.16 ng/mL/1 gm total protein) significantly decreased the level of COX-2 as compared to UV injured group at *p* < 0.05 (Fig. 3).

#### Prostaglandin E2 (PGE-2) tissue level

UV irradiation significantly increased level of PGE-2 (125.6 ± 10.88 pg/mL/1 gm total protein) as compared to control (30.89 ± 3.29 pg/mL/1 gm total protein) at *p* < 0.05. Coriander oil in both cream (64.77 ± 8.729 Pg/mL/1 gm total protein) and CEONLC nanoemulgel formulation (61.29 ± 18.02 pg/mL/1 gm total protein) significantly decreased the level of PGE-2 as compared to UV injured group at *p* < 0.05 (Fig. 3).

#### Collagen tissue level

UV irradiation resulted in decrease in collagen level (5.23 ± 0.94 ng/ml/1 gm total protein) compared to control group (19.11 ± 3.47 ng/mL/1 gm total protein) at *p* < 0.05. Coriander oil in both cream (9.77 ± 0.67 ng/mL/1 gm total protein) and CEONLC nanoemulgel formulation (10.64 ± 1.61 ng/mL/1 gm total protein) significantly increased the level of collagen as compared to UV injured group at *p* < 0.05 (**Fig. ****3**).

#### Elastin tissue level

UV irradiation significantly increased skin elastin content (80.68 ± 9.29 ng/mL/1 gm total protein) compared to control group (13.03 ± 1.33 ng/mL/1 gm total protein) at *p* < 0.05. Coriander oil in both cream (37.88 ± 9.49 ng/mL/1 gm total protein) and CEONLC nanoemulgel formulation (38.26 ± 8.09 ng/mL/1 gm total protein) significantly decreased skin elastin content as compared to UV injured group at *p* < 0.05 (Fig. 3).

#### Matrix metalloproteinase 1 (MMP-1) expression level

The mRNA expression of MMP-1 was significantly increased in UV injured group (5.59 ± 0.89) compared to the control group (1.017 ± 0.01) at *p* < 0.05. Coriander oil in both cream (1.96 ± 0.28) and CEONLC nanoemulgel formulation (1.67 ± 0.33) significantly decreased MMP-1 level as compared to UV injured group at *p* < 0.05, moreover CEONLC nanoemulgel formulation showed a non-significant difference than the control group (Fig. 3).

#### Activator protein 1 (AP-1) and c-Jun N-terminal kinase (JNK) expression level

**Figure 4:**
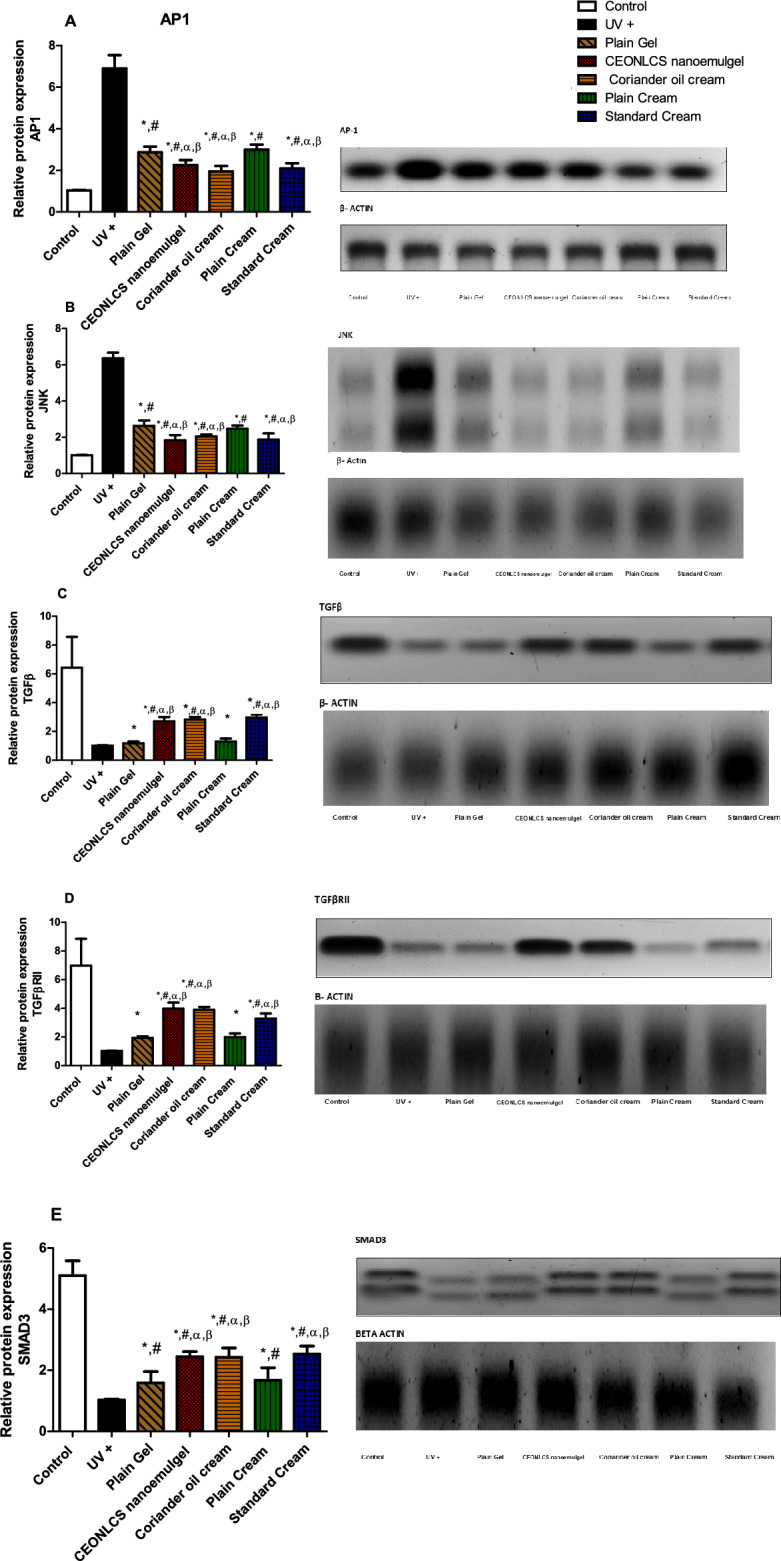
Effect of plain cream, plain gel, Coriander oil cream formula, CEONLC nanoemulgel formulation, and standard cream treatment on protein expression of (**A**) AP-1, (**B**) JNK, (**C**) TGF*β*, (**D**) TGF*β*RII, and (**E**) SMAD3 in skin homogenates of photoaged mice. Each result represents the mean value for 8 mice ± SD of the mean. Statistical analysis was carried out by one-way ANOVA followed by Tukey’s multiple comparison test. *Statistically significant from the normal control group at *p* ≤ 0.05, ^#^statistically significant from the UV injured group at *p* ≤ 0.05, α statistically significant from the plain cream base group at *p* ≤ 0.05, and *β* statistically significant from the plain gel base group at *p* ≤ 0.05.

UV irradiation-induced expression of transcription factor AP-1 and JNKsas demonstrated by their increased level in UV injured group (AP-1: 6.9 ± 0.64; JNK: 6.36 ± 0.32) as compared to control group (AP-1: 1.04 ± 0.017; JNK: 1.01 ± 0.01). Coriander oil in both cream (AP-1: 1.95 ± 0.27; JNK: 2.05 ± 0.11) and CEONLC nanoemulgel formulation (AP-1: 2.26 ± 0.23; JNK:1.83 ± 0.29) reversed UV-inducible Ap-1 and JNK expression as manifested by significantly decreased levels of AP-1 and JNK, compared to UV injured group at *p* < 0.05 (Fig. 4).

#### Transforming growth factor *β* (TGFβ), Transforming growth factor *β* receptor II (TGFβII) and SMAD Family Member 3 (SMAD3) expression level

UV irradiation impaired TGFβ signaling leading to a significant decrease in TGFβ, TGFβII, and SMAD3 expression in UV injured group (TGFβ: 1.01 ± 0.02; TGFβII: 1.02 ± 0.02; SMAD3:1.03 ± 0.03) as compared to the control group (TGFβ: 6.43 ± 2.15; TGFβII: 6.98 ± 1.88; SMAD3: 5.1 ± 0.48) at *p* < 0.05. Coriander oil in both cream (TGFβ:2.83 ± 0.17; TGFβII: 3.89 ± 0.19; SMAD3:2.43 ± 0.30) and CEONLC nanoemulgel formulation (TGFβ: 2.70 ± 0.30; TGFβII: 3.97 ± 0.42; SMAD3: 2.44 ± 0.17) restored TGFβ signaling as manifested in significantly increased expression level of TGFβ, TGFβII, and SMAD3 as compared to UV injured group at *p* < 0.05. There was no significant difference in the all-previous biomarkers tissue levels among coriander oil cream, CEONLC nanoemulgel formulation, and standard cream (Fig. 4).

#### The external appearance of the dorsal skin in the different experimental groups

**Figure 5:**
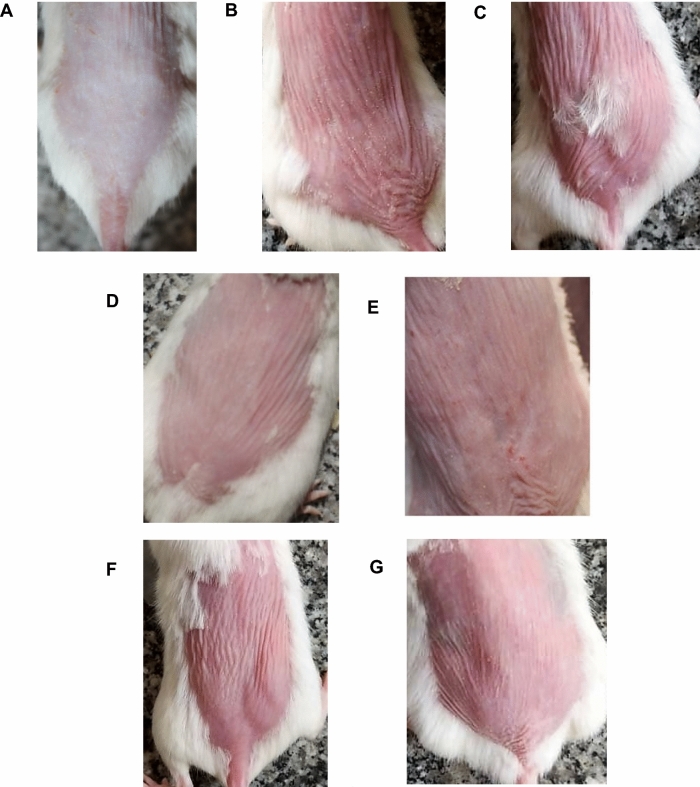
Photographs for the dorsal side skin of mice: (**A**) normal skin, (**B**) UV+, (**C**) Plain gel, (**D**) CEONLCS, (**E**) Corriander oil cream, (**F**) Plain cream, (**G**) Standard cream.

Coriander oil in both cream and CEONLC nanoemulgel formulation improve the skin appearance as compared to UV-injured group (Fig. 5).

## Discussion

There is a strong link between people's health and their appearance. As a result, the healthier an individual is, the more beautiful skin appears, and worsening of health will have a detrimental impact on appearance due to skin degradation^16^. In addition, there was always a relationship between skin aging and oxidative stress as well as other intrinsic and extrinsic factors. In recent years, the development of cosmetic medications for improving skin appearance or reducing skin aging has gotten a lot of attention. Several research has been carried to assess the anti-aging properties of various plants^17,18^. Essential oils (EOs) and their components have long been listed in traditional medicine and aromatherapy as ingredients in popular cosmetics and cosmeceuticals with reported anti-aging potentiality^19^. Aromatic plants from the Apiaceae family produce, from their vegetative and reproductive organs, essential oils inside oil ducts, known as vittae^20^. Given the availability and high EO, Apiaceous oils are exploitable for different naturally-derived pharmaceutical applications^21^. An interesting perspective is their utilization in skin preparations, aiming to be eco-friendly alternatives to synthetic cosmetics^20,22^. Recently, nanomaterials-based cosmetic products also known as nanocosmetic formulations are used to be innovative solutions to overcome the drawbacks of conventional cosmetic products. Many nanoproducts were developed in the field of cosmetic industry such as metallic nanoparticles (silver nanoparticles and gold nanoparticles), solid lipid nanoparticles, liposomes, nanoemulsions and nanogels^23^. These nanocosmetic products offers many advantages such as better entrapment of cosmetic ingredient (such as cosmetic oils), enhanced penetration, improved efficacy, and higher stability of the final cosmetic formulations^24^. The market sales of nanocosmetic formulations is predicted to exceeds US$55.3 billion by the current year^25^. Nanocosmetic products are developed to treat different problems for example skin aging and hyperpigmentation also hair damage and fall^23^. Nanoformulation encapsulating oils offers the potential of higher penetration to the deeper layers of the skin; therefore, antiaging agents like vitamin E, retinol, and vitamin C showed higher efficacy when delivered in the form of nanoformulation^26,27^.In this study, the essential oils of the four selected apiaceous plants (fennel, anise, cumin, and coriander) were tested in vitro for their anti-aging activities. The skin aging-related enzyme assays revealed that coriander oil showed the highest inhibitory activity of tyrosinase, elastase, hyaluronidase, and collagenase compared to fennel, anise, and cumin. The chemical composition of coriander essential oil that was analyzed by gas chromatography with flame ionization and mass spectrometry detection revealed that linalool was the highest percentage composition in the essential oil (81.29%). Linalool has been for a long time incorporated in formulations such as cosmetics, perfumes, soaps as well as household cleaners for its specific fragrance^28^.

High doses of UV light can overwhelm the natural anti-oxidant defense mechanisms of the skin which can lead to significant damage of most of the cellular components by free radicals, therefore the use of essential oils continues to trend upwards as they are safe and possess several therapeutic properties. Therefore, we hypothesized that coriander oil may possess in vivo anti-aging properties. Our results showed that coriander oil formulations possessed potent antiaging potential through the inhibition of various cell signaling pathways, down-regulation of the mRNA expression of MMP-1 as well as a potent antioxidant and anti-inflammatory properties. To examine the cellular mechanisms through which coriander can improve UV-induced damage, we assessed the levels of collagen, elastin, COX2, PGE2, MDA, SOD, AP-1, JNK, TGFB, TGFB-RII, and SMAD3 along with the expression of MMP-1. Our results showed that the mRNA expression of MMP-1 and the protein concentration of AP-1 and JNK were significantly elevated which in turn led to a significant decrease in collagen levels in the UV- injured group compared to the control group. Treatment with coriander oil led to a significant decrease in the expression of MMP-1 and protein concentrations of AP-1 and JNK which finally led to a significant improvement in the levels of collagen compared to the UV- injured group. Our results are in agreement with a study carried by Hwang et.al 2014, which showed that treatment with coriander leaf extracts significantly inhibited the activity of transcription factor AP-1, expression of MMP-1, and pro-collagen type 1 in-vivo and in-vitro^9^.

UV radiation causes a significant increase in the production of ROS, which directly activates MMP-1 and increases its expression. It also leads to the excitation of the MAPK family (composed of JNK, P38, and extra-cellular signal-regulated kinase). JNK then binds to the c-fos to form the transcription factor AP-1 which is involved in regulating MMP-1 expression, thereby increasing the breakdown of collagen^29^. Results showed that TGFB, TGFBRII, and SMAD3 were significantly decreased in the UV- injured group compared to the control group. Meanwhile, treatment with coriander oil formulations significantly increased these parameters. These results are in agreement with the study conducted by Park et.al 2018 where treatment with *Eucalyptus globulus* extract was able to enhance TGFβ/Smad signaling thereby inhibiting MMP-1 expression^30,31^.

Transforming growth factor-β (TGF-β) is a cytokine that plays an important role in regulating the biosynthesis and degradation of extracellular matrix proteins^32^. It binds to TGF-β type II receptor (TβRII), which in turn phosphorylates TGF-β type I receptor (TβRI). This phosphorylation activates transcription factors SMAD2 and SMAD3. When either of these activated transcription factors combines with Smad4 they form heteromeric Smad complexes that are translocated into the nucleus causing upregulation of collagen production and downregulation of MMPS^9,33^.

In aged skin, AP-1 induced by ROS was found to inhibit the TGF-β signaling pathway^34^. Our study showed a significant increase in the levels of COX2 and PGE2 in the UV-injured group compared to the control group. Treatment with coriander oil preparations significantly decreased the levels of COX2 and PGE2 compared to the UV-injured group. These results are in agreement with the study that was conducted to assess the effect of another natural compound; orange peel extract against UV-induced photoaging where it was found to possess potent anti-inflammatory properties^35^. UV radiation results in the extensive production of ROS which activates p38 MAPK thereby enhancing COX2 expression and hence the production of PGE2^29,36^.

Elastin levels were significantly elevated in the UV- injured group compared to the control group. Groups treated with coriander oil preparation showed a significant reduction in the levels of elastin compared to the UV- injured group. UV radiation activates the elastin promotor leading to the accumulation of poorly organized elastin (solar elastosis) in the skin. These results are in agreement with Weihermann et.al, 2017 who suggest that skin photoaging can be characterized by a reduction in the concentration of functional elastin and an increase in non-functional elastin fiber production^37^. These results can be attributed to the fact that coriander oil is a potent antioxidant that significantly improves damage caused by UV-induced oxidative stress. Terpenoids are mostly present in the form of essential oils in higher medicinal plants and exists in several families including Umbelliferae, Compositae, Rutaceae and Labiatae^38^. Recent reports showed that oxygenated terpenes, either in essential oils or plant extracts, are added to health care products owing to their cosmetic potential^39^. Our Analysis revealed that oxygenated monoterpenes represented the highest proportion of Coriander, Anise, Fennel and Cumin essential oils. Additionally, previous data revealed also that linalool, as the major constituent of coriander oil, exhibited significant antioxidant potential which has been reflected in its preventive effect against low-dose UVB-induced ROS generation and subsequent antioxidant enzyme depletion^40^. This could be explained through inhibiting UVB-mediated overexpression of several inflammatory mediators as: TNF-α, IL-6, IL-10, and COX-2 in skin cells, also decreasing the overexpression of MMP-2 and MMP-9^40^.

The cosmetic industry has been forced by contemporary customer demands and international legislation to seek new active components from natural eco-friendly and safe renewable sources. So, the results of the current study confirmed that coriander essential oil formulations possessed the highest collagenase, elastase, tyrosinase, and hyaluronidase inhibitory activities. Additionally, in vivo anti-aging assessment of the developed conventional as well as nano-based pharmaceutical formulations revealed astonishing results. Where they exhibited inhibition of various cell signaling pathways and down-regulation of the mRNA expression of MMP-1. In conclusion, coriander essential oil is considered a promising natural source in the cosmetics industry for skin as a result of both antiaging potentiality and odorous characters. But this necessitates further clinical studies, which will be the inevitable goal of our future work.

## Materials and methods

### Plant material and essential oils extraction

The fruits of Apiaceae (*Foeniculum vulgare* L., *Pimpinella anisum* L., *Coriandrum sativum* L., and *Cuminum cyminum* L.) were obtained from Agricultural Research Center, Cairo, Egypt in August 2019. Permission to collect fruits of Apiaceae were obtained from Agricultural Research Center, Cairo, Egypt. Fruits were authenticated by Eng. Theres Labib, Consultant of Plant Taxonomy at Ministiry of Agriculture, Egypt. Voucher specimens (20–08-2018) were kept in the herbarium of Departement of Pharmacognosy and Natural Products, Faculty of Pharmacy, Menoufia University, Menoufia, Egypt. Essential oils (EO) were extracted from the four dried Apiaceous fruits by the hydro-distillation method using the Clevenger apparatus^14^. The distilled essential oils were dried over anhydrous sodium sulphate, filtered, and stored in a sealed vial at − 20 °C until analyzed.

### In vitro screening of selected oils on skin aging-related enzyme activities

#### Determination of collagenase inhibitory activity

The assay employed was done as previously reported^41^, with slight modifications. In a 96-well microplate, 20 μL of enzyme solution (0.8 units/mL) was added, followed by 20 μL of various concentrations of tested substances (1000–7.81 g/mL) and finally 20μL of Tricine buffer (50 mM, pH 7.5). EO samples were dissolved in (DMSO 5% (v/v) in Tricine buffer) to varying concentrations. 40 μL of the substrate (2 mM) was added after a 15-min. incubation time at 37 °C. Using a microplate reader, the absorbance was determined at 335 nm immediately after adding substrate and then continuously for 20 min. (Tecan, USA). Water was used as the negative control. As a positive control, EGCG was used. The percentage of collagenase inhibitin (%) = $\left( {1 - \frac{S}{C}} \right) \times 100$, where ‘S’ is the corrected absorbance of the samples containing collagenase inhibitor (the enzyme activity in the presence of the samples), and ‘C’ is the corrected absorbance of controls (the enzyme activity in the absence of the samples). The IC_50_ value was defined as the concentration of the sample to inhibit 50% of collagenase under the assay conditions.

#### Determination of elastase inhibitory activity

The anti-elastase activity was measured using the method of^42^, with minor modifications. In 96-well plates, 25 μL of 0.1 M HEPES buffer (pH 7.5), tested samples (1000–7.81 g/mL), and elastase enzyme (1 g/l) were mixed. EO samples were dissolved in (DMSO 5% (v/v) in HEPES buffer) to varying concentrations. After 20 min. at room temperature, 100 μL of the substrate N-methoxysuccinyl-Ala-Ala-Pro-Val-p-nitroanilide (1 mM) was added to the plates, which were then incubated for another 40 min. at 25 °C. Absorbance was read at 405 nm using a Tecan Infinite 500 spectrophotometer (USA). The positive control was done using elafin. The percentage inhibition was calculated as follows: % of inhibition = (A _control_/A_tested sample_)/ A_control_) × 100. Where A_control_ is the absorbance without adding of inhibitor and A sample is an absorbance after adding oils or Elafin.

#### Determination of tyrosinase inhibitory activity

The tyrosinase inhibition assay was carried out using L-DOPA as the substrate and followed the method reported by^43^. Firstly, 685 μL of phosphate buffer (0.05 M, pH 6.5), 15 μL of tyrosinase enzyme (2500 U /mL), 200 μL of samples solution (7.81–1000 µg/mL) and 100 μL of 5 mM/L L-DOPA were added to a 96 plate. EO samples were dissolved in (DMSO 5% (v/v) in phosphate buffer) to varying concentrations. The absorbance was immediately measured. As a positive control, kojic acid was utilized. Each experiment was repeated three times. The percentage inhibition was calculated as follows: % of inhibition = (A_control_/A_tested sample_)/ A_control_) × 100. Where A_control_ is the absorbance without adding of inhibitor and A sample is an absorbance after adding oils or standard.

#### Determination of hyaluronidase inhibitory activity

Hyaluronidase inhibitory activity of oils was evaluated by a spectrometric method^44^. The samples were tested at concentrations of 7.81–1000 µg/mL μg/mL. Oils (50 μL) were incubated with hyaluronidase enzyme solution (10 μL) at 37 °C for 10 min then added calcium chloride (12.5 mM, 20 μL) and re-incubation at 37 °C for 10 min. In the reaction mixture, sodium hyaluronate (50 L) was added and incubated at 37 °C for 40 min, followed by the addition of sodium hydroxide (0.9 M, 10 L) and sodium borate (0.2 M, 20 L) and incubation at 100 °C for 3 min. PDMAB (50 μL, 67 mM) was added to the reaction mixture and incubated at 37 °C for 10 min. Absorbance was measured at 585 nm. Percent enzyme inhibition was calculated as given below, compared to the control. Sodium aurothiomalate was used as the reference standard. The percentage inhibition was calculated as follows: % of inhibition = (A_control_/A_tested sample_)/ A_control_) × 100, where A_control_ is the absorbance without adding of inhibitor and A sample is an absorbance after adding oils or standard. The IC_50_ value, a concentration giving 50% inhibition of hyaluronidase activity, was determined by interpolation of concentration–response curves.

### Essential oil analysis

The EO were analyzed using a combination of gas chromatography and mass spectrometry (GC–MS, Shimadzu GCMS-QP2010, Tokyo, Japan) equipped with Rtx-5MS fused bonded column (30 m × 0.25 mm i.d. × 0.25 µm film thickness) (Restek, USA) equipped with a split–splitless^45^. The first column temperature was retained at 45 °C for 2 min. (isothermal) and programmed to 300 °C at a rate of 5 °C/min., and kept constant at 300 °C for 5 min. (isothermal). The injector temperature was 250 °C. Helium carrier gas flow rate was 1.41 mL/min. The following conditions were used to record all of the mass spectra: (equipment current) filament emission current, 60 mA; ionization voltage, 70 eV; ion source, 200 °C. The split mode was used to inject diluted samples (1% v/v) (split ratio 1: 15). The study was performed using MS spectra, which were compared to the NIST library's spectra as well as data from the literature. The components were identified based on a comparison of their relative retention times and mass spectra with those of standards, Wiley and NIST library data of the GC–MS system, and literature data^46^.

### Preparation of Coriander essential oil-loaded Lipid nanoparticles (CEOLNs)

Coriander essential oil-loaded Solid Lipid Nanoparticles (**CEOSLNs**) were prepared by using cocoa butter as a solid lipid and L-*α*-lecithin as a surfactant. Coriander essential oil-loaded Nanostructured Lipid Carriers **(CEONLC)** were prepared by using, solid lipid (cocoa butter), liquid lipids (olive oil (or) sesame oil), and surfactant (lecithin)^15^. For the preparation of **CEOSLNs**, 200 mg of cocoa butter was melted at 40 °C, coriander EO was added gradually to the melted lipid and an aqueous phase containing 130 mg of lecithin (15 mL) heated to 40 °C was poured into the lipid phase containing (cocoa butter and Coriander essential oil) under high shear homogenization of 24,000 rpm for 15 min with UltraTurrax equipment (T25, IKA, Labortechnik, Denmark). The whole nanoemulsion system was directly sonicated by using a bath sonicator (Elmasonic S80 H, Elma Hans Schmidbauer GmbH & Co, Singen, Germany) for 30 min to avoid aggregation of nanoparticles during cooling. Preparation of CEONLCwas similar to the previous methods with the only difference of adding 50 mg of olive oil to 150 mg of cocoa butter before melting. Blank SLNs and blank NLC were prepared following the previous method without the addition of the oil extract. The composition of the prepared nanosystems is demonstrated in Table 3.

**Table 3:** Formulation components of the prepared CEOLNs.

Formula	Components			
	Cocoa butter (mg)	Olive oil (mg)	L-*α*-lecithin (mg)	Coriander Oil (mg)
Blank SLNs	200	0	130	0
Blank NLC	150	50	130	0
CEOSLNs	200	0	130	50
CEONLC	150	50	130	50

### Preparation of nanoemulgel containing CEONLC

CEONLC formulations were gelled using xanthan gum as a gelling agent according to the method described by^47^. For the preparation of the gel base of xanthan gum, 3 mg of the gum was added to 100 ml of deionized water and stirred continuously to form a gel base, followed by pH adjustment with triethanolamine. The prepared CEONLC in the form of nanoemulsion was mixed with xanthan gum gel base by magnetic stirring at 400 rpm for about 20 min until the homogenous distribution of CEONLC through the nanoemulgel matrix. The oil was poorly water-soluble, we mixed an equal amount of oil extract with Tween-80 as a solubilizing agent before mixing with the gel base.

### Characterizations of CEOLNs

#### Nanoparticle size, polydispersity index (PDI), and zeta potential

The particle size, polydispersity index, and zeta potential of the prepared **CEOLNs** were measured using (Malvern Zetasizer Nano ZS90, UK) and all measurements were performed in triplicate at room temperature^48,49^.

#### Transmission electron microscopy (TEM):

The surface morphology of **CEOLNs** was measured using TEM imaging (JEM 2010, Jeol, Peabody, MA, USA) operating at an acceleration voltage of 200 kV. A liquid aliquot of lipid nanoparticles dispersions was placed on a formvar copper grid and the sample was dried at room temperature before imaging^50^.

### In-vivo evaluation of the anti-aging activity of Coriander essential oil formulations

#### Animals and experimental design

All the methods were performed in accordance with the relevant guidelines and regulations following the recommendations in the ARRIVE guidelines^51^. The animal protocols used in this study were reviewed and approved based on the ethical procedures and scientific care of animals set by the ethics committee at October University for Modern Sciences and Arts (MSA, approval no. PH11/EC11/2020F on 11/2020) and by the recommendations of the National Institute for Health Guide for the care and treatment of laboratory animals. Fifty-six adult 8 week old female Swiss Albino mice weighing 25–30 g were obtained from the National Scientific Research Centre in Giza, Egypt. They had free access to food and water and were acclimatized for one week before commencing with the experimentation. Mice (n = 56) were first assigned to either a no radiation (Control) (n = 8) or a UV radiation (n = 48) group. The UV radiation group was then further divided into six groups of eight mice each; untreated group, a gel base treated group, a cream base treated group, a coriander EO cream treated group, a CEONLC-nanoemulgel formulation, and C-Topic (standard group, commercial preparation with Vitamin C as the main active ingredient) treated group.The choice of the standard cream was based on the fact that Vitamin C possesses potent anti-oxidant properties in addition to its ability in stimulating the production of collagen, thereby protecting the skin against damage induced by UV-radiations^52^. Mice fur was removed using a hair removal cream for sensitive skin so as to expose the dorsal skin and avoid any injuries that could happen while shaving, this process was repeated as often as needed to ensure that the dorsal skin is exposed and that the topical treatments were properly applied. Photoaging was induced using UV-radiaiton where the UV lamp (UVB lamp, Idea Boeki, Tokyo, Japan ) was placed 30 cm above the dorsal skin of the hairless mice and the minimal erythemal dose (MED) was determined by exposing the skin to different doses of UV radiation and detecting the formation of erythema after 24 h. Each mouse was placed in a fitted cage to minimize movement during irradiationWrinkles and photoaging were induced by exposing the dorsal skin to 1 MED (100 mJ/cm2) three times weekly for six weeks^53^. Topical treatments were initiated after the induction of wrinkles where 0.25 gm of each formula was applied twice daily for five weeks to the designated group^54^. At the end of the experiment, photoaging was assessed macroscopically by observation the dorsal skin. The animals were sacrificed, and skin tissue samples were acquired for biochemical analysis.

#### Biochemical analysis

The collected dorsal skin samples (10 mg) were minced and homogenized (1:10 ratio) in lysis buffer (50 mm Tris/HCl, 150 mm sodium chloride, 1.0% NP-40, 0.5% sodium deoxycholate, 0.1% SDS, pH 7.6). After homogenization, the samples were centrifuged for 20 min. at 20,000 rpm and the supernatant was separated and stored in Eppendorf tubes at -80˚C until assayed.

##### Measurement of oxidative stress biomarkers

The concentration of MDA and SOD (Biodiagnostic, Diagnostic and research reagents, Egypt) were determined in the tissue lysate colorimetrically by a UV/Visible spectrophotometer(Schimadzu spectrophotometer 2401 UV/Visible, Japan)^55,56^.

##### Measurement of Cyclooxygenase 2, Prostaglandin E2, collagen and elastin by ELISA

The concentrations of cyclooxygenase 2, prostaglandin E2, collagen, and elastin in the tissue lysate were determined using commercially available ELISA kits (Cyclooxygenase 2, ELISA Kit (MBS020842), MyBioSource, USA; Prostaglandin E2, ELISA kit, (CSB-E07966m), CUSABIO, USA; and Elastin ELISA Kit (E-EL-R0004), Elabscience, USA according to the manufacturer’s instructions.

##### Gene expression and qRT-PCR

RNA was extracted using RNeasy Mini Kits (QIAGEN, Venlo, Netherlands) according to the manufacturer’s protocol. RT-qPCR was performed by use of SYBR® Green dye (iScriptTM One-Step RT-PCR Kit, Bio-Rad, California, United States) on Rotor-Gene Q real-time PCR cycler (QIAGEN, Venlo, Netherlands). *β*-actin gene was used as a control gene. The forward and reverse primers used for amplification of Matrix metalloproteinase 1 (MMP-1) and *β*-actin are listed in Table 4.

**Table 4:** The forward and reverse primers used for amplification of Matrix metalloproteinase 1 (MMP-1) and *β*-actin.

Primer		The sequence of nucleotides (nt)	Size (nt)
MMP-1	Forward	5′ CTA TTC TGT CAG CAC TTT GG 3′	20
	Reverse	5′ CAG ACT TTG GTT CTC CAA CTT 3′	21
*β*-actin	Forward	5′ GAC CTT CAA CAC CCC AGC CA 3′	20
	Reverse	5′ GTC ACG CAC GAT TTC CCT CTC 3′	21

##### Western blotting

The expression of AP1, JNK, TGF*β*, TGFBRII, and SMAD3 were evaluated using western blot analysis. The proteins prepared from the skin tissues of different study groups were separated by 4–20% sodium dodecyl sulfate–polyacrylamide gel electrophoresis (SDS-PAGE) for 1 h, and the resolved proteins were transferred to a nitrocellulose membrane. The blot was run for 7 min at 25 V to allow protein bands to transfer from the gel to membrane using Bio-Rad Trans-Blot Turbo. Each membrane was incubated separately with the primary antibody: anti-AP-1 (1:500 dilution, GW21143; Sigma-Aldrich, Saint Louis, MO, USA ), anti-JNK(1:500 dilution, MA5-15881; Invitrogen, USA), anti-TGFβ (1:500 dilution, PA5-86215; Invitrogen, USA), anti-TGFBRII (1:1000 dilution,PA5-35076; Invitrogen, USA), anti SMAD3 (1:1000 dilution, PA5-34774; Invitrogen, USA), and anti-actin (1:1000 dilution, MA1-744; Invitrogen, USA) overnight at 4˚C. The blot was rinsed 3–5 times for 5 min with TBST buffer, the membranes were then washed with washing buffer and incubated with horseradish peroxidase (HRP)-conjugated goat anti-rabbit IgG (1:1000 dilution, Goat anti-rabbit IgG- HRP-lmg Goat mab -Novus Biologicals) solution against the blotted target protein for onehr at room temperature. The blot was rinsed 3–5 times for 5 min with TBST buffer. Finally, the membrane blots were developed using a Chemiluminescence Reagent (ClarityTM Western ECL substrate - BIO-RAD, USA cat#170-5060). The chemiluminescent signals were captured using a CCD camera-based imager. Image analysis software was used to read the band intensity of the target proteins against the control sample after normalization by β actin on the Chemi Doc MP imager (Supplementary File 1).

### Statistical analysis

Data were analyzed using the GraphPad Prism 6.0 statistical program (GraphPad Software, Inc.), and statistical differences between groups were evaluated using one-way analysis of variance. *p* < 0.05 was considered to indicate a statistically significant difference.

### Ethical approval

The study was conducted based on the ethical procedures and scientific care of animals set by the ethics committee at October University for Modern Sciences and Arts (MSA), Egypt, (approval no. PH11/EC11/2020F on 11/2020) and by the recommendations of the National Institute for Health Guide for the care and treatment of laboratory animals.

## Supplementary Information

Supplementary Information.
