# Correction: Coriander (*Coriandrum sativum* L.) essential oil and oil-loaded nano-formulations as an anti-aging potentiality via TGFβ/SMAD pathway

**DOI:** 10.1038/s41598-026-51904-1

**Published:** 2026-05-28

**Authors:** Mohamed A. Salem, Eman G. Manaa, Nada Osama, Nora M. Aborehab, Mai F. Ragab, Yusuf A. Haggag, Magda T. Ibrahim, Dalia I. Hamdan

**Affiliations:** 1 Department of Pharmacognosy and Natural Products, Faculty of Pharmacy, Menoufia University, Gamal Abd El Nasr St., Shibin Elkom, 32511 Menoufia Egypt; 2 Clinical Pharmacy Department, Shibin Elkom Teaching Hospitals, Gamal Abd El Nasr St., Shibin Elkom, 32511 Menoufia Egypt; 3 Biochemistry Department, Faculty of Pharmacy, Menoufia University, Gamal Abd El Nasr St., Shibin Elkom, 32511 Menoufia Egypt; 4 Department of Biochemistry, Faculty of Pharmacy, October University for Modern Sciences and Arts (MSA), Giza, 12451 Egypt; 5 Pharmacology Department, School of Life and Medical Sciences, The University of Hertfordshire Hosted By Global Academic Foundation, New Administrative Capital, Cairo, Egypt; 6 Department of Pharmaceutical Technology, Faculty of Pharmacy, Tanta University, Tanta, Egypt; 7 Department of Pharmacognosy, Faculty of Pharmacy, Al-Azhar University, Cairo, Egypt

Correction to: *Scientific Reports* 10.1038/s41598-022-10494-4, published online 21 April 2022

The original Article contained errors. Due to an error during figure assembly of Fig. 4, the western blot panel for JNK in Fig. 4B was duplicated from the AP1 panel in Fig. 4A. The JNK panel in Fig. 4B has been replaced. The corresponding underlying image in the supplementary material has been corrected.

The original Fig. 4 and accompanying legend appear below:

**Fig. 4:**
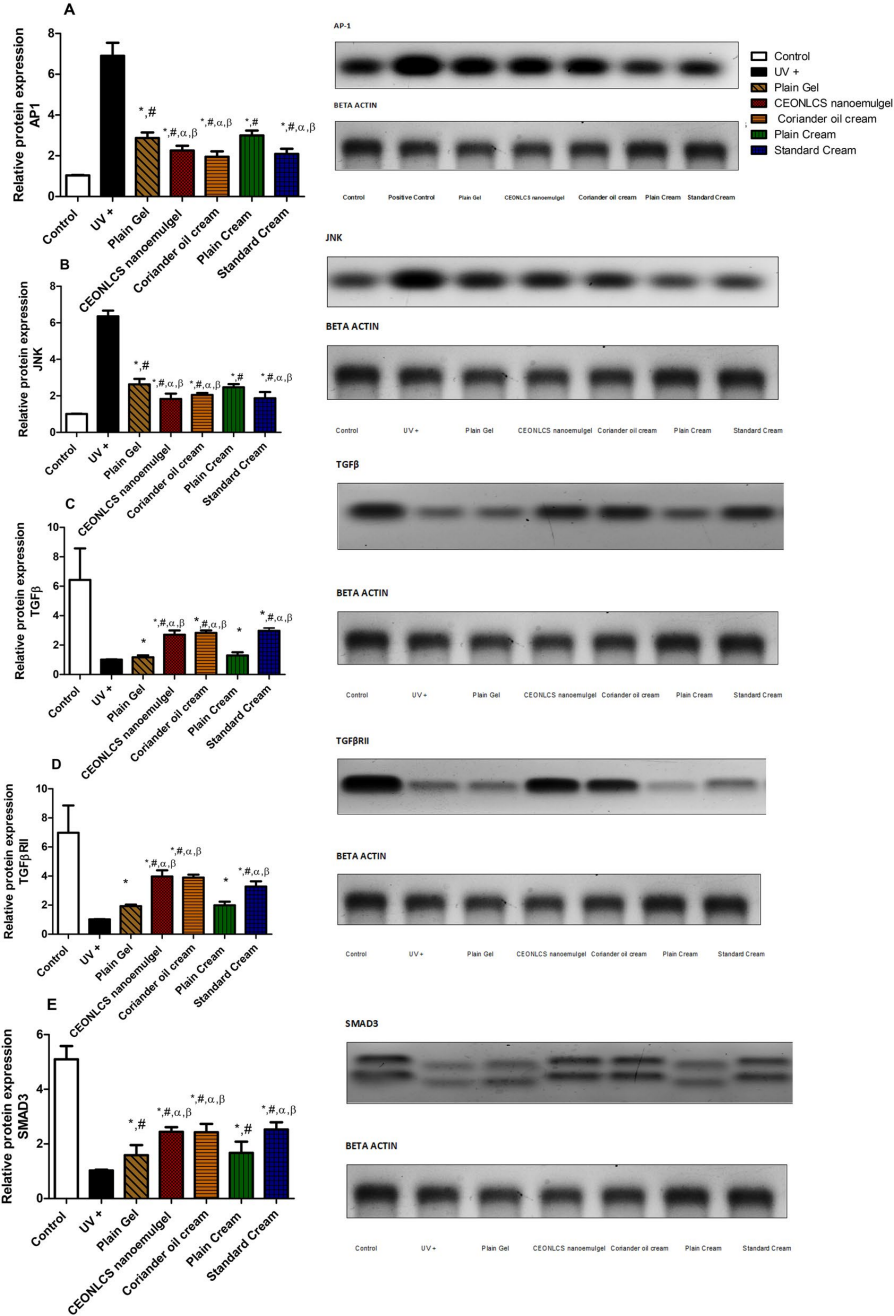
Effect of plain cream, plain gel, Coriander oil cream formula, CEONLC nanoemulgel formulation, and standard cream treatment on protein expression of (**A**) AP-1, (**B**) JNK, (**C**) TGF*β*, (**D**) TGF*β*RII, and (**E**) SMAD3 in skin homogenates of photoaged mice. Each result represents the mean value for 8 mice ± SD of the mean. Statistical analysis was carried out by one-way ANOVA followed by Tukey’s multiple comparison test. *Statistically significant from the normal control group at *p* ≤ 0.05, ^#^statistically significant from the UV injured group at *p* ≤ 0.05, α statistically significant from the plain cream base group at *p* ≤ 0.05, and *β* statistically significant from the plain gel base group at *p* ≤ 0.05.

In addition, due to an error during figure assembly of Fig. 5, the image of Fig. 5C shows a different photograph of the same animal depicted in Fig. 5F. The image in Fig. 5C has been replaced with an animal from the correct treatment group. The original Fig. 5 and accompanying legend appear below:

**Fig. 5:**
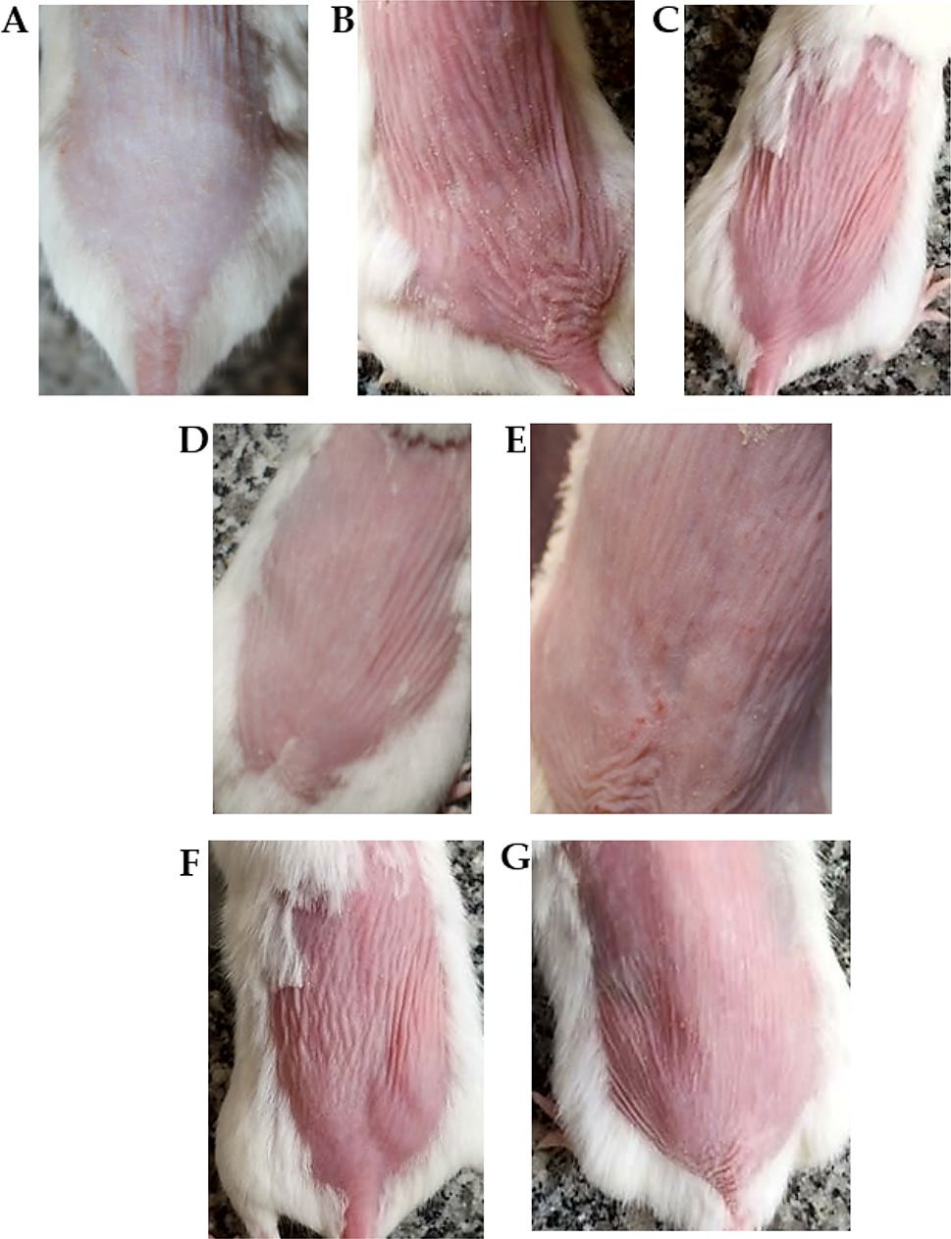
Photographs for the dorsal side skin of mice: (**A**) normal skin, (**B**) UV+ , (**C**) Plain gel, (**D**) CEONLCS, (**E**) Corriander oil cream, (**F**) Plain cream, (**G**) Standard cream.

The original Article has been corrected.

## Supplementary Information

Supplementary Information.

