# Supplementary figures and images for: Coriander (Coriandrum sativum L.) essential oil and oil-loaded nano-formulations as an anti-aging potentiality via TGFβ/SMAD pathway
Source: Sci Rep. 2022 Apr 21;12:6578. doi: 10.1038/s41598-022-10494-4 (PMC9023561; doi:10.1038/s41598-022-10494-4)

Supplementary file: Original Western blot gel

Beta actin


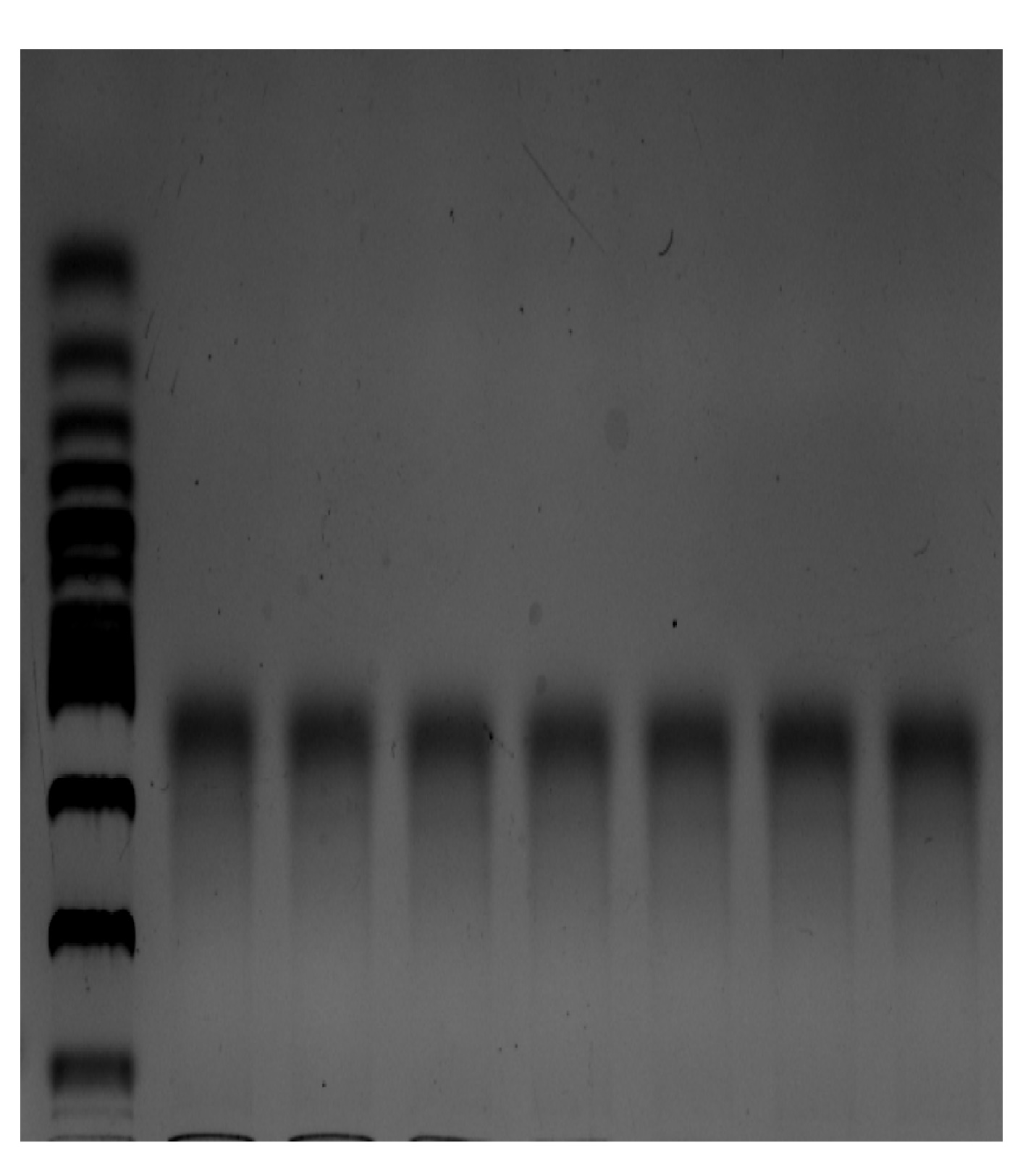


Ap-1


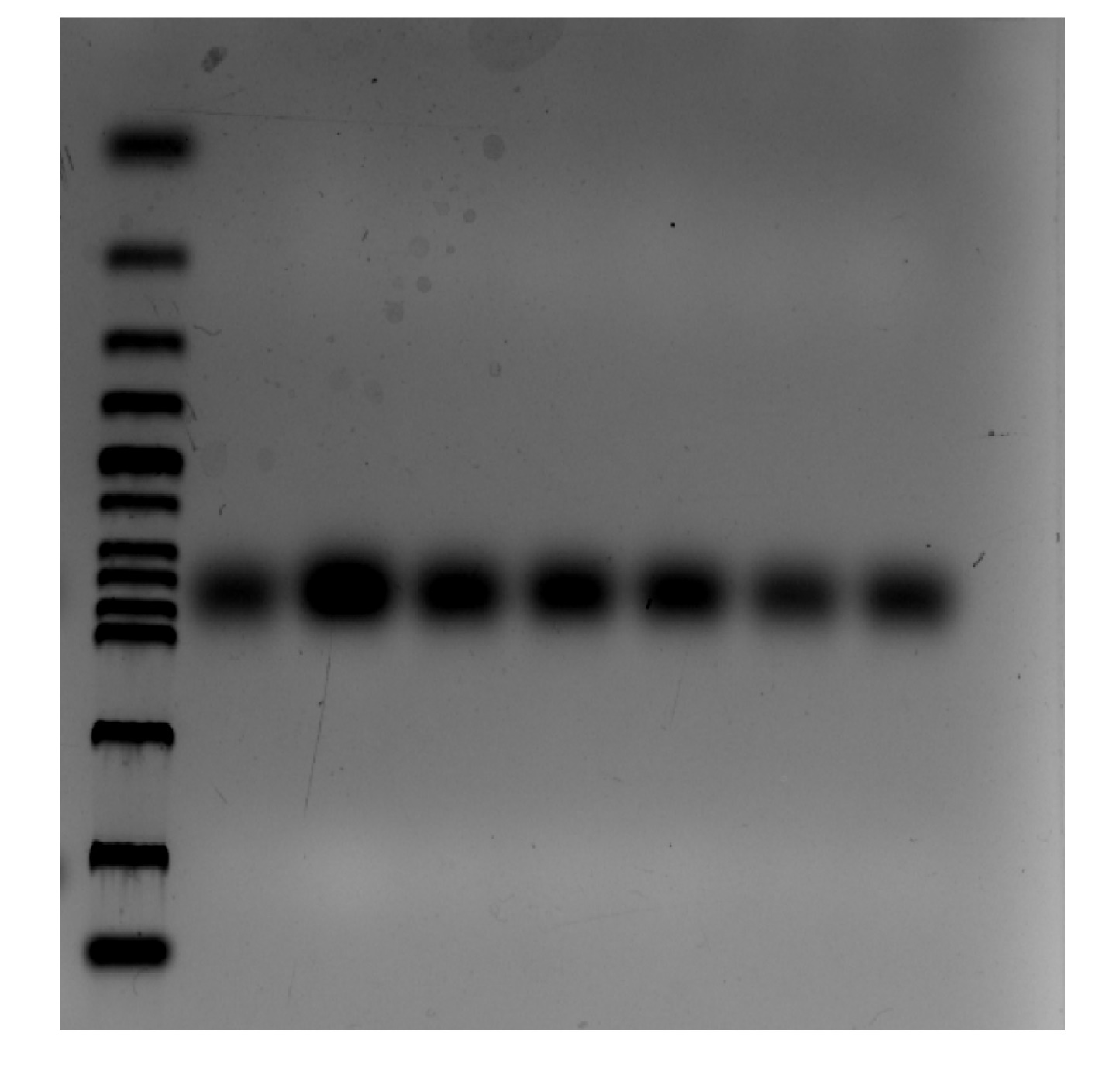


JNK


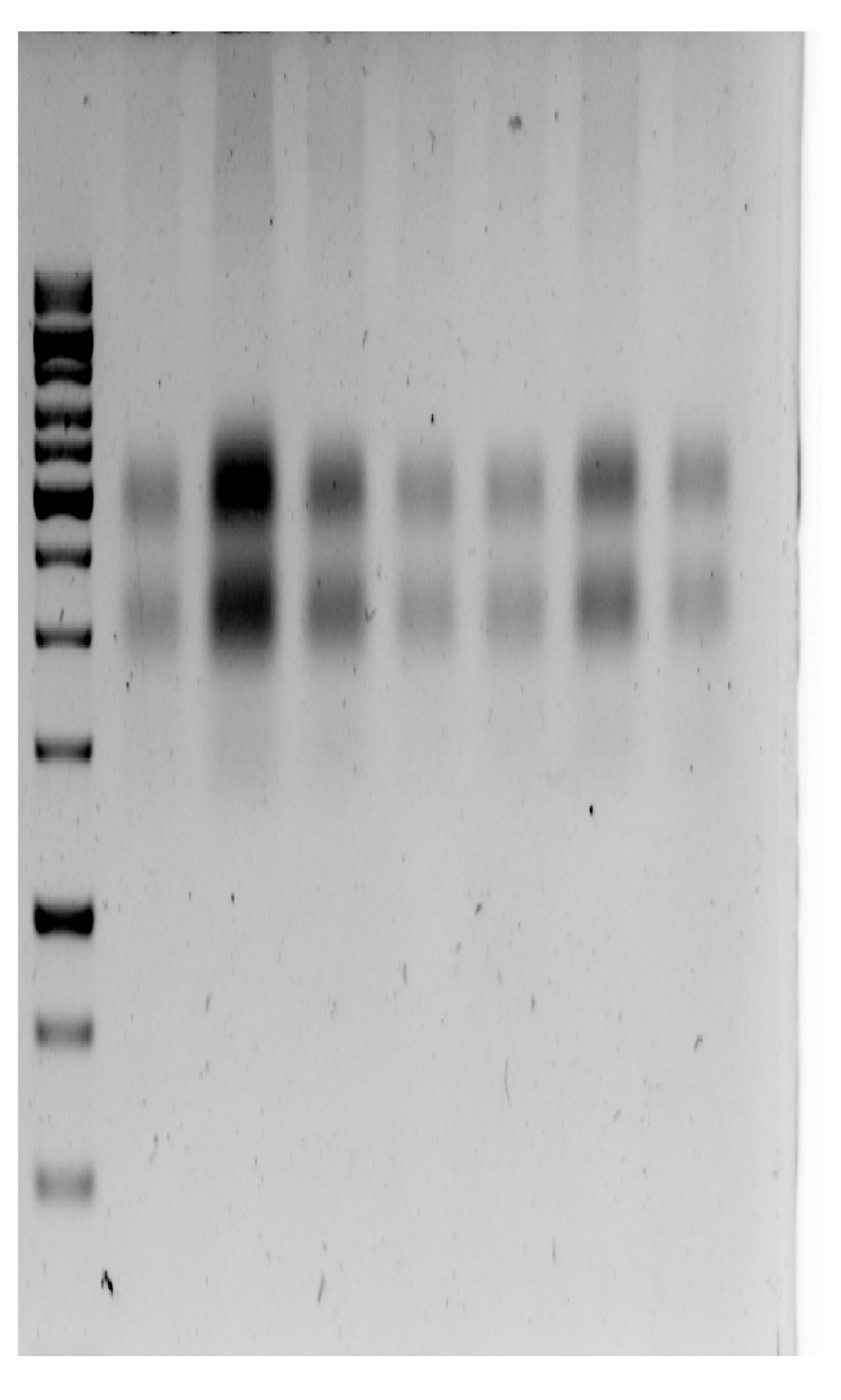


TGFβ


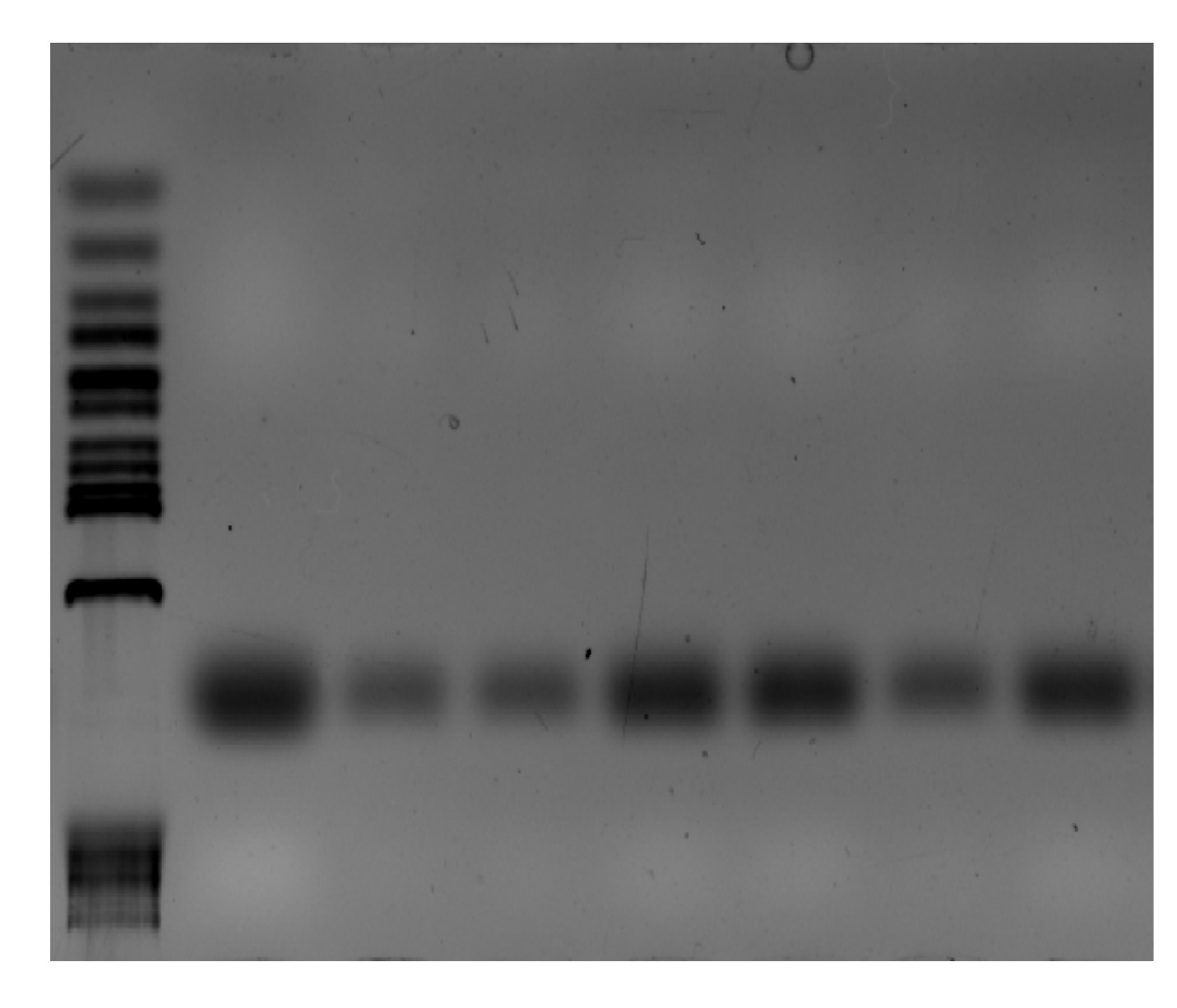


TGFβRII


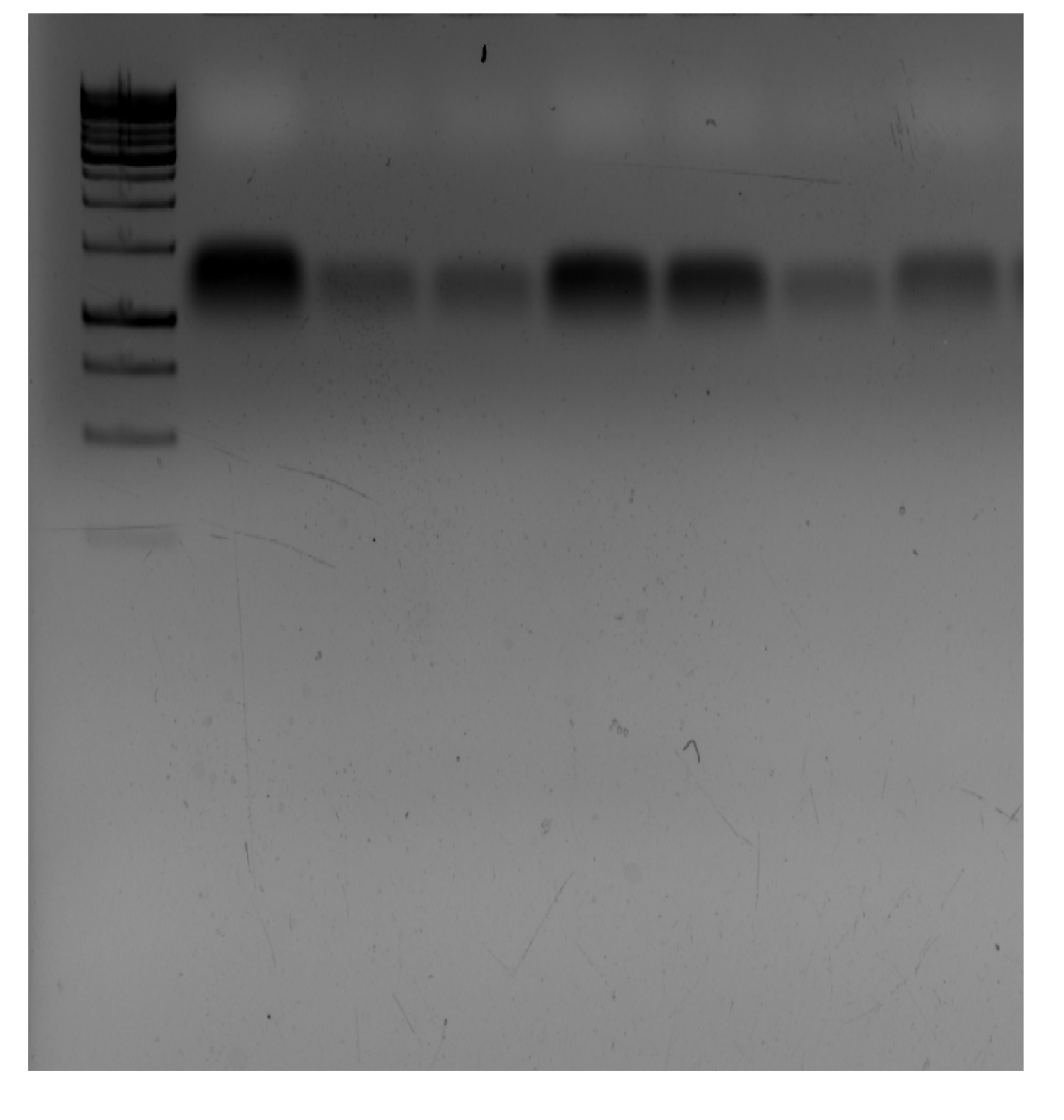


SMAD3


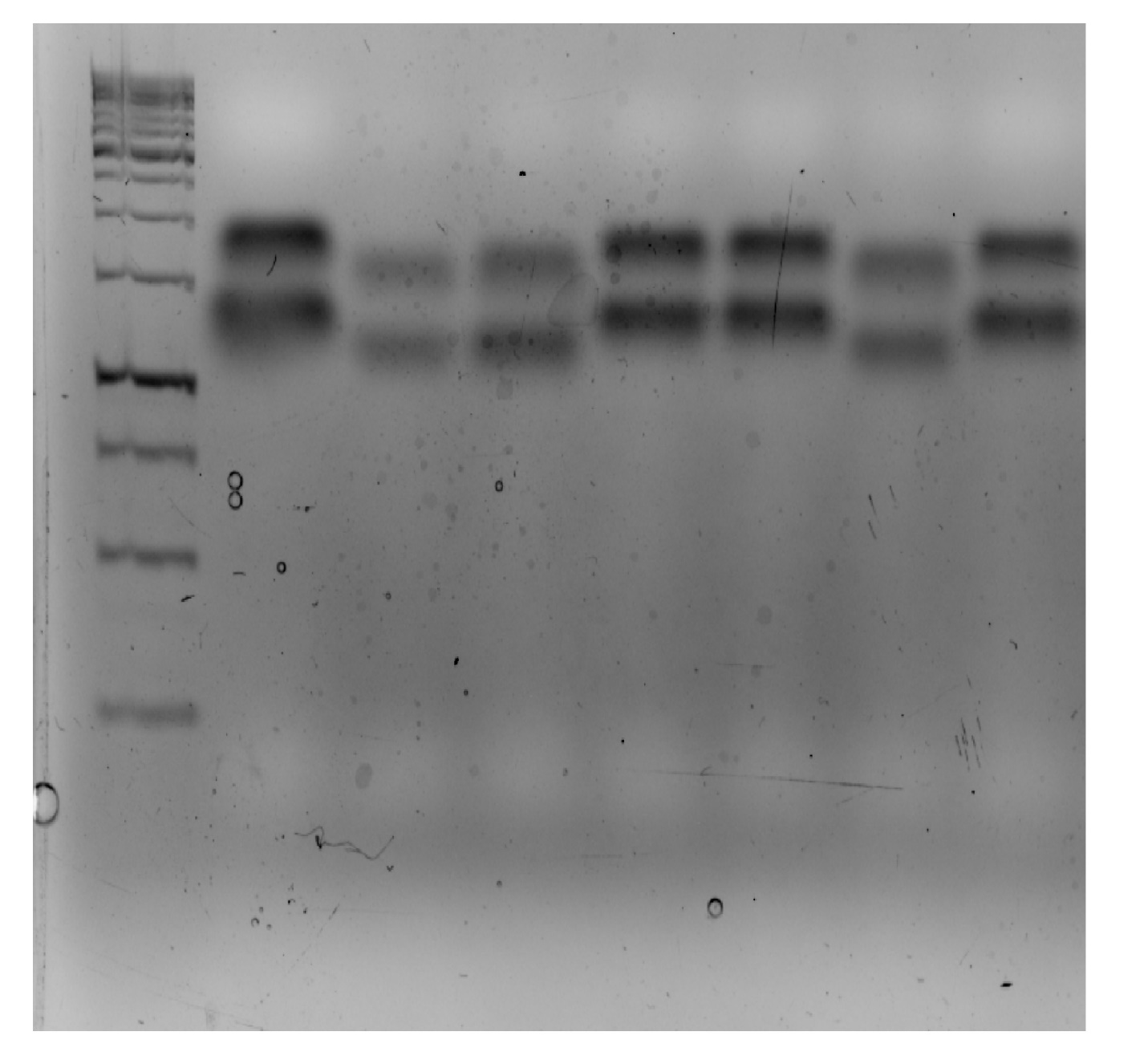

Supplement: Supplementary file 1 — Supplementary Information. [file 41598_2022_10494_MOESM1_ESM.docx]
