# Supplementary figures and images for: Correction: Coriander (Coriandrum sativum L.) essential oil and oil-loaded nano-formulations as an anti-aging potentiality via TGFβ/SMAD pathway
Source: Sci Rep. 2026 May 28;16:16584. doi: 10.1038/s41598-026-51904-1 (PMC13219796; doi:10.1038/s41598-026-51904-1)

Supplementary file: Original Western blot gel

Beta actin


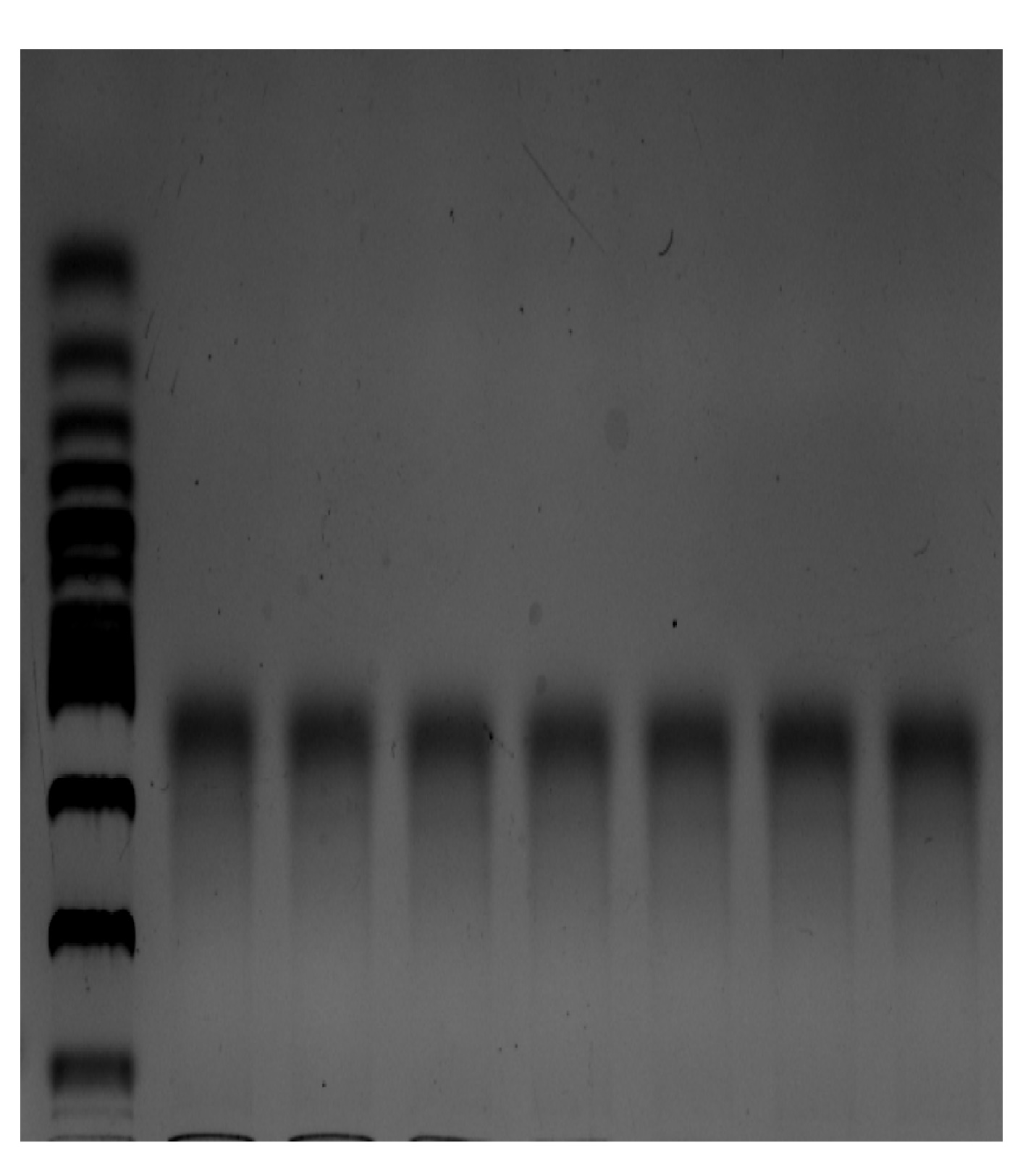


Ap-1


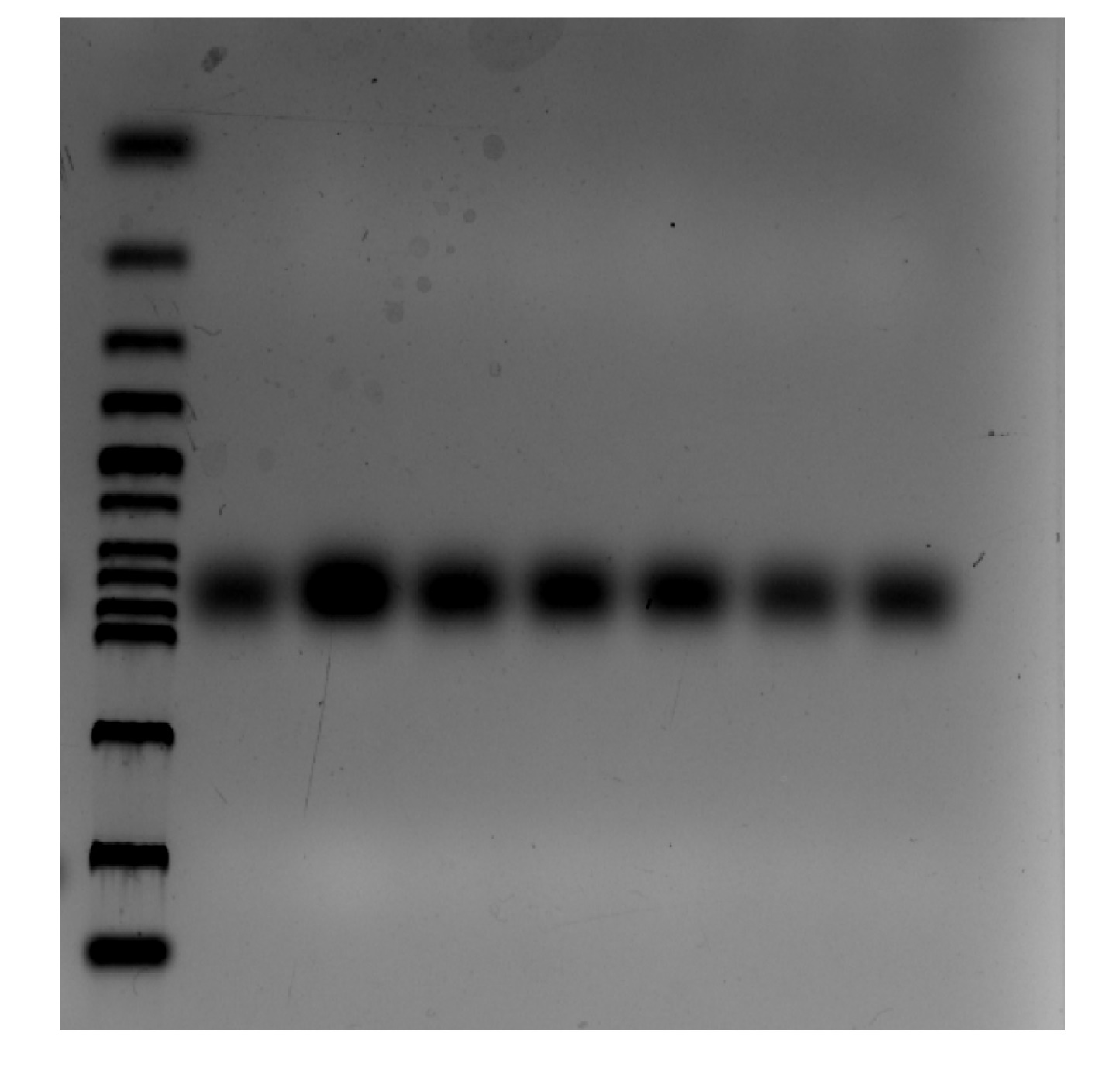


JNK


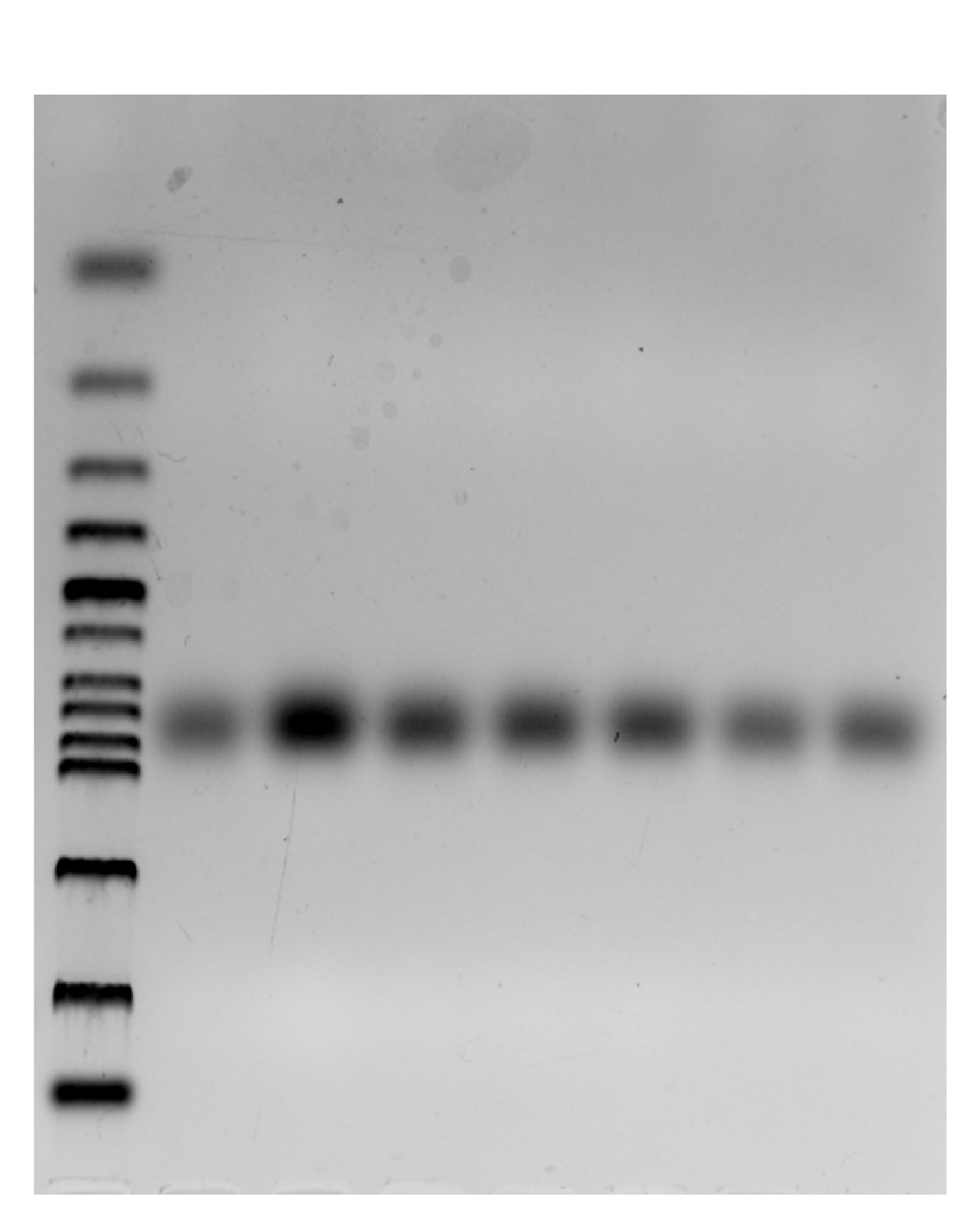


TGFβ


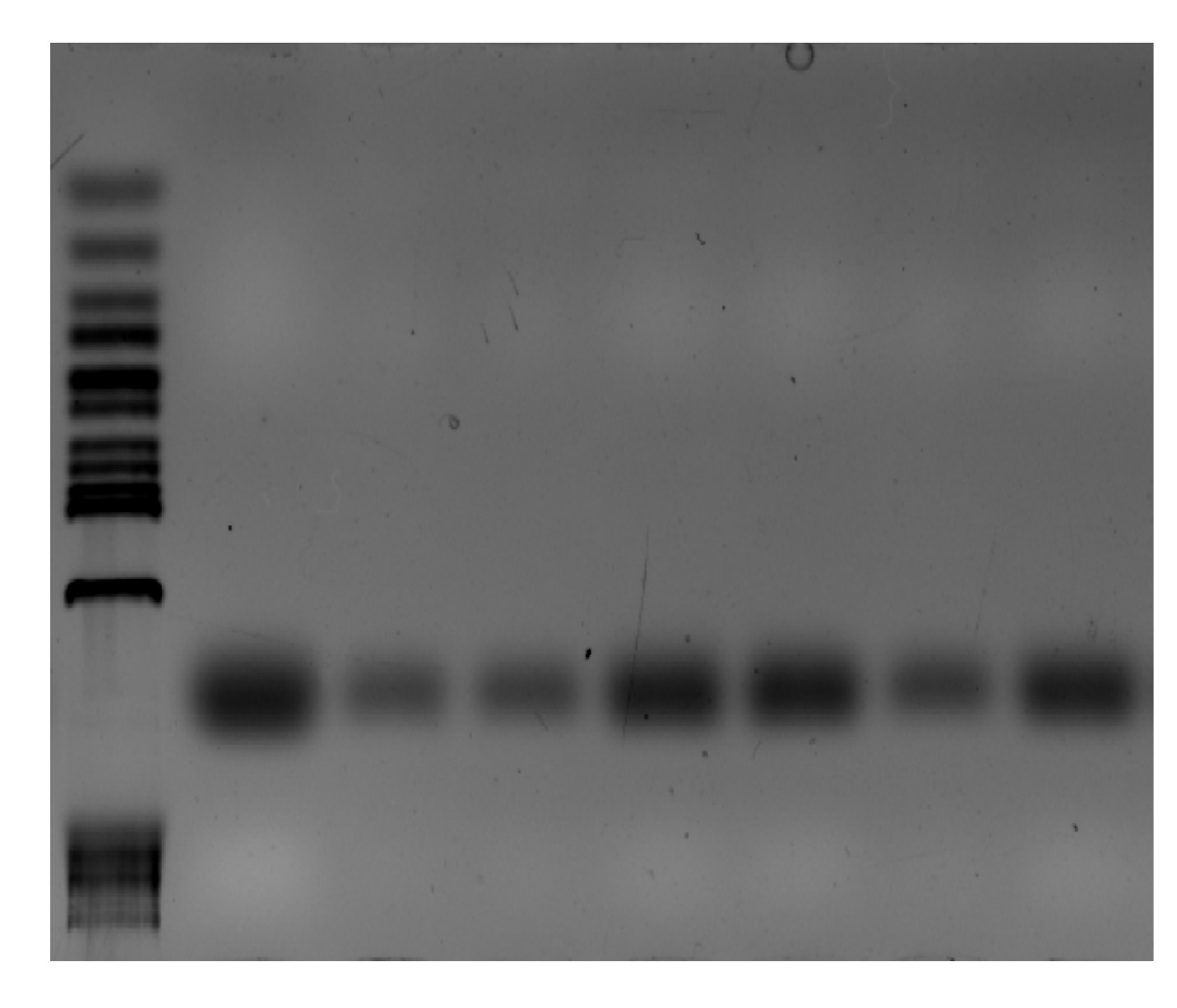


TGFβRII


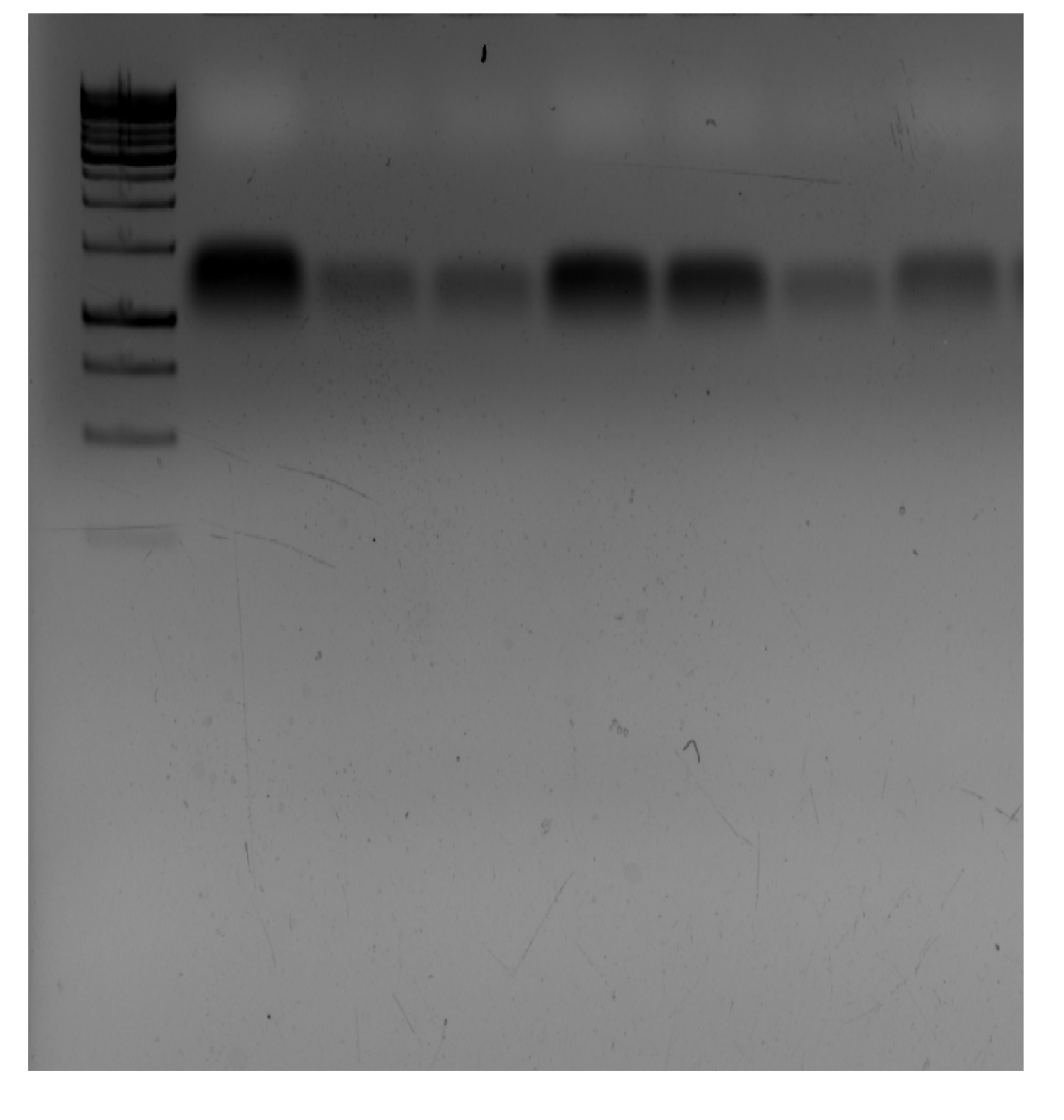


SMAD3


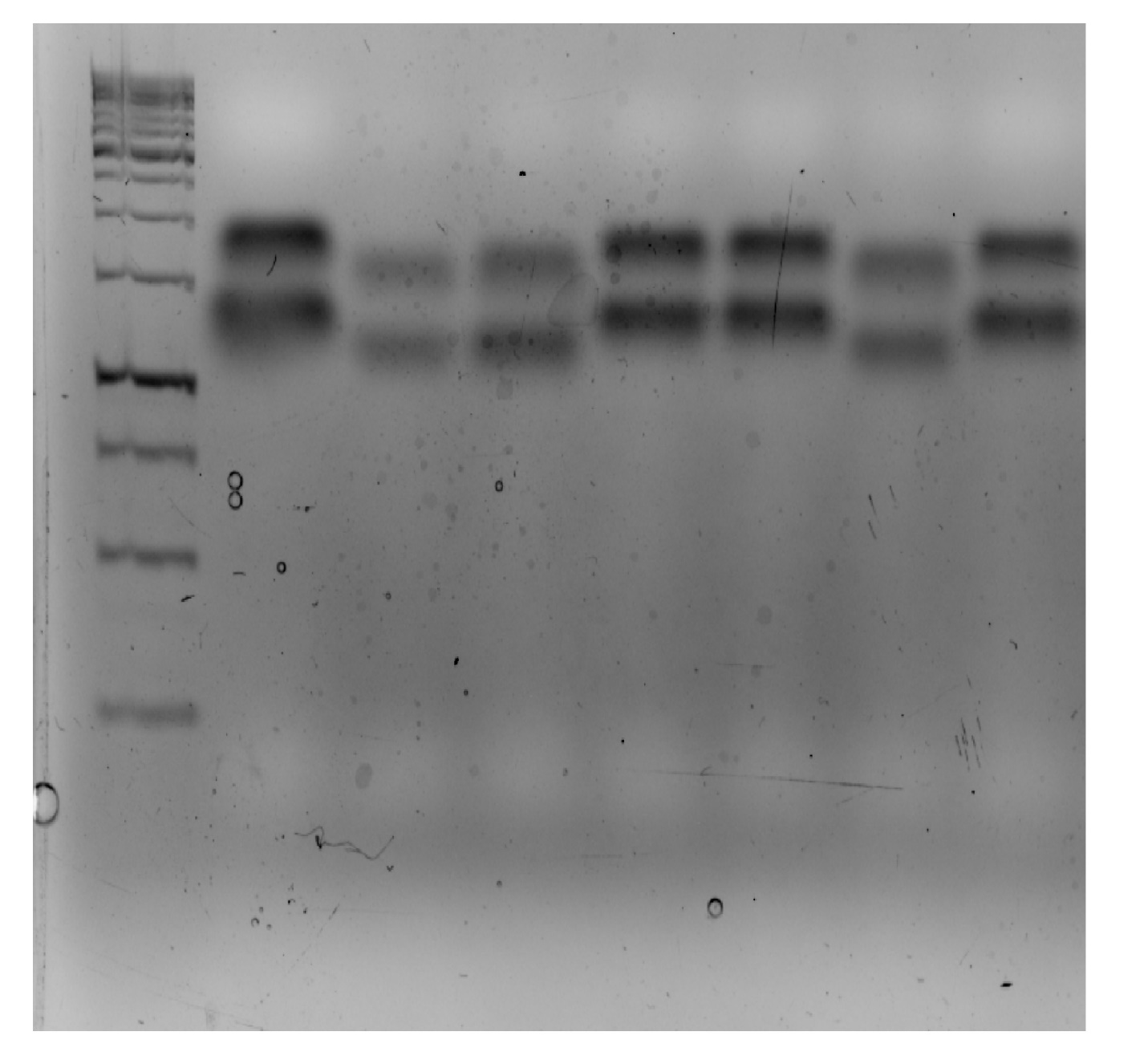

Supplement: Supplementary file 1 — Supplementary Information. [file 41598_2026_51904_MOESM1_ESM.docx]
